# Different depths of food restriction and high‐fat diet refeeding in mice impact host obesity and metabolic phenotypes with correlative changes in the gut microbiota

**DOI:** 10.1002/mco2.641

**Published:** 2024-07-17

**Authors:** Jiaqi Xu, Huangru Xu, Feiyan Yang, Yawen Xie, Fangfang Cai, Siyu Mao, Min Lu, Hongqin Zhuang, Zi‐Chun Hua

**Affiliations:** ^1^ The State Key Laboratory of Pharmaceutical Biotechnology and Department of Neurology of Nanjing Drum Tower Hospital School of Life Sciences and The Affiliated Hospital of Nanjing University Medical School, Nanjing University Nanjing China; ^2^ Faculty of Pharmaceutical Sciences Xinxiang Medical University Xinxiang China; ^3^ Changzhou High‐Tech Research Institute of Nanjing University and Jiangsu TargetPharma Laboratories Inc. Changzhou China

**Keywords:** energy intake, enteritis, FR15%–Re, gut microbiota, lipid metabolism

## Abstract

Overweight and obesity affect almost 2 billion adults worldwide, and food restriction (FR) is commonly used to reduce body fat. Whether refeeding (Re) after FR at different ages and to different degrees leads to overweight and its possible mechanisms are uncertain. In this study, adult and young mice were both restricted to 15% and 40% of their casual food intake, and then were fed 60% high‐fat chow (FR15%–Re, FR40%–Re), whereas the control groups(CON) consumed high‐fat or normal food throughout, respectively. The results of the study suggest that mild FR‐heavy feeding may lead to more significant abnormal fat accumulation, liver damage, and increased recruitment of intestinal inflammatory factors and immune cells in mice of different ages and involves multiple types of alterations in the gut microbiota. Further fecal transplantation experiments as well as serum and liver enzyme‐linked immunosorbent assay experiments preliminarily suggest that the link between lipid metabolism and inflammatory responses and the gut microbiota may be related to the regulation of the gut and live by Lipopolysaccharides(LPS) and Peroxisome Proliferator‐Activated Receptor‐Alpha(PPAR‐α). In addition, our study may also serve as a reference for studying obesity prevention and treatment programs at different ages.

## Graphical abstract

FR15%–Re has long‐term negative effects on the host, including abnormal fat accumulation, liver damage, and intestinal inflammation, which are associated with the gut microbiota. ELISA experiments have shown that impaired intestinal barrier function in mice leads to the entry of LPS into the bloodstream and the liver and the downregulation of PPAR‐α in the liver.

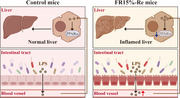

## 1 INTRODUCTION

Overweight and obesity affect almost 2 billion adults worldwide, with more than half of them being obese.^1^ Through a variety of energy‐restricted dietary strategies, people can reduce body weight and enhance their health.^2^ Among them, fasting and food restriction (FR) are popular methods for losing weight.^3^ FR is the practice of lowering daily caloric intake by 15%−40% without risking starvation.^4^ Nevertheless, studies have shown that fasting and FR are associated with reduced chronic and systemic inflammation.^5^, ^6^, ^7^ Noteworthy, FR also causes a significant alteration in the gut microbiota linked to energy harvest.^8^, ^9^, ^10^

It is well acknowledged that gut microbiota and health are critically influenced by food quantity (managing food intake) and have been considered as a key mediator linking diet and host physiology.^3^, ^11^, ^12^, ^13^ First, in the gut–diet interaction, the balance of proinflammatory and anti‐inflammatory reactions in the gut is directly influenced by the microbiota.^13^, ^14^ Second, feeding depth, or the regularity with which food is consumed, is critical.^15^, ^16^ The host eating depth is disrupted and the composition of the gut microbiota is changed as a result of FR,^17^ and whether refeeding (Re) can restore the microbiota oscillations becomes an interesting question. Third, modifications to the microbiota alter gene expression not only locally in the intestine but also in distant organs like the liver.^18^, ^19^ Previous studies have reported that dietary restriction altered the gut microbiota into a Lactobacillus (L.)‐dominated structure, and an isolated Lactobacillus strain helped lower inflammation.^20^, ^21^ However, a significant issue in the treatment of obese people is the weight regain after weight loss and how it alters gut microbiota.^2^

Compensatory weight gain at the end of FR will lead to greater attention to diet and dietary restriction.^22^ Weight regain after weight loss may play a critical role in illness development.^23^ The degree of FR–Re in the FR–Re protocol, as well as the gut–diet interaction at the time of food availability, may have a multifaceted influence on the people.^22^, ^23^ Consistently, similar behavior in mice has been noticed in numerous studies. A recent study demonstrates a connection between mice's weight gain following FR and a more effective metabolic phenotype that involves a distinctive gut microbiota structure for energy harvesting.^24^ However, the effects of FR and Re depth on host metabolic profiles, gut microbiota, and inflammation are not well understood. It has been shown that disturbances in the intestinal microbiota and disruption of the barrier function lead to the production of lipopolysaccharides (LPS), an endocytotoxin produced by intestinal bacteria, which plays an important role in the development of liver disease.^24^ More needs to be learned about the link between lipid metabolism and inflammatory responses and gut microbiota involved in this dietary strategy.

C57BL/6J mice are now recognized as a standard strain in physiology, immunology, and other studies. They are sensitive to high‐fat chow and become obese relatively quickly after high‐fat feeding. The modeling time of this experiment was long, and male mice were used in order to avoid the interference of the estrous cycle of female mice on the experimental results. In this study, we focused on the extent and phenotype of body weight recovery and preliminary mechanisms in mice restricted to food at different ages at the end of different restrictions. To determine the effects of altering the depth of FR and Re on host physiology and gut microbiota effectiveness, we implemented the same two FR regimens on 12‐week‐old adult and 6‐week‐old young mice and examined their metabolic phenotypes, gut microbiota, and correlation analyses to explore the long‐term effects of these two depths of FR–Re on hosts of different ages. In this study, mice were subjected to varying degrees of FR. Each mouse was restricted to 85 and 60% of its ad libitum intake before being fed a high‐fat diet. The study found that FR15%–Re mice experienced significantly different physiologic changes, including fat accumulation, liver inflammation, enteritis, and changes in energy intake. In addition, the gut microbiota of FR15%–Re mice remained significantly different from that of FR40%–Re and both controls over a long period of time. We describe changes in gut microbiota and metabolic phenotypes in FR and Re, infer that putting mild dietary restriction to an end after a high‐fat diet may lead to altered gut microbiota and more unfavorable metabolic phenotypes, and preliminarily explore their interconnections.

## 2 RESULTS

### 2.1 FR15%–Re mice exhibited altered metabolic outcomes regardless of age

**FIGURE 1:**
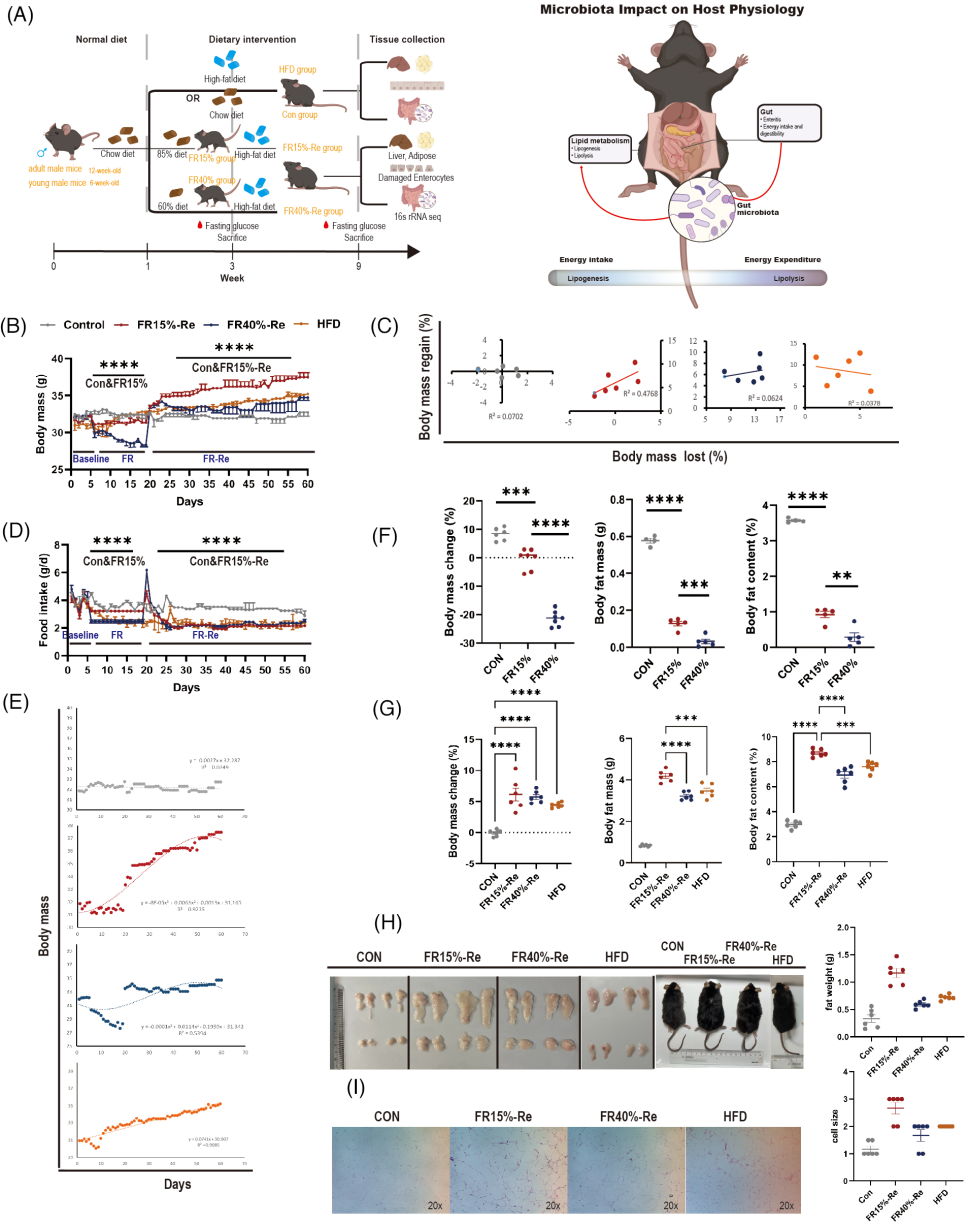
Observations on the metabolic effects of food restriction and refeeding. (A) CON, the animals were fed ad libitum throughout the experiment; HFD, the animals were fed 60% high‐fat chow throughout the experiment; FR15%–Re, FR40%–Re, the animals were restricted by 15 and 40% of ad libitum food intake, respectively, for 2 weeks and followed by high‐fat diet refeeding for 6 weeks. (B) Body mass of the food‐restricted mice refed with a high‐fat diet. (C) Correlations between body mass lost during 2 weeks of food restriction and body mass regain of mice refed with a high‐fat diet for 6 weeks. (D) The food intake of mice in each group was recorded daily. (E) The functional relationship and fitting curve for the four groups’ changes in body mass. (F) Body mass change, body fat mass and body fat content of the food‐restricted mice. (G) Body mass change, body fat mass, and body fat content of the food‐restricted mice refed with a high‐fat diet. (H) Body fat and mouse morphology of the food‐restricted mice refed with a high‐fat diet. (I) Hematoxylin and eosin (H&E) staining of white adipose tissue. Data were represented as mean ± SEM. *n* = 6 mice in each group. *p < 0.05, **p < 0.01, ***p < 0.001, ****p < 0.0001.

The study was conducted on 48 adult (12 weeks old) and 48 young (6 weeks old) male C57BL/6J mice, which were randomly assigned to four groups (CON, HFD, FR15%–Re, FR40%–Re) of 12 mice each, for a total of eight groups. How the two different FR–Re diets affected physiological and metabolic responses in mice of different ages was investigated (Figure 1A). Details of these methods are shown in the section on animal testing in the *Materials and Methods* section.

First, we examined how body mass was altered in 48 12‐week‐old mice by two levels of the FR–Re diet. At 1 week of basal measurement, there was no difference in body weight among the four groups of mice. After 2 weeks of FR, mice in the two food‐restricted groups lost significant weight, with the FR40% group losing more weight than the FR15% group. When FRs ended, body weights recovered significantly. At the end of 6 weeks of high‐fat Re, there were significant differences between the body weights of the groups, with the FR15%–Re group instead rebounding more significantly than the FR40%–Re group and weighing more than the mice in the HFD group that had been continuously high‐fat fed during the same time period, whereas there were no significant differences between the FR40%–Re group and the CON group that had been continuously fed a regular chow diet (Figure 1B). Next, we examined intergroup differences in body weights of 48 6‐week‐old mice receiving the same experimental treatment. Consistently, FR and Re had a significant effect on body weight in the FR15%–Re group. At the end of 6 weeks of high‐fat Re, the FR15%–Re group was significantly lower than the CON and HFD groups, whereas there was no significant difference between the FR40%–Re and CON groups (Figure S1A). There was a significant positive correlation between the rate of weight loss and weight regain in the FR15%–Re group in adult mice (*R* ^2^ = 0.4768; Figure 1C) and a significant negative correlation in the FR15%–Re group in young mice (*R* ^2^ = 0.213; Figure S1B). In addition, we observed significant differences between the FR15%–Re group and the HFD, CON, and FR40%–Re groups throughout the 6‐week period of Re (from 4 to 10 weeks) in both age groups of mice, with significantly higher body mass return in the FR15%–Re group of adult mice, consistent with the function *Y* = −8E−0.5 × 3 + 0.0063 × 2 + 0.0013*x* + 31.165 expression (*R* ^2^ = 0.9235; Figure 1E). The FR15%–Re group of young mice had significantly lower body mass return than the other groups, consistent with the expression of the function *Y* = −*Y* = −6E−0.7 × 3 − 0.0012 × 2 + 0.116*x* + 20.829 (*R* ^2^ = 0.8575; Figure S1C). These findings suggest that FR15%–Re, but not FR40%–Re, causes paradoxical changes in body mass, regardless of age.

FR significantly affects body fat content. First, we euthanized six mice in each of eight experimental groups of adult and juvenile mice at the end of 2 weeks of FR. We found that body fat and histologic characteristics of adult mice were altered with two levels of FR–Re diet. The results showed that during the basal measurement period, body fat mass and content were significantly reduced in adult mice on both depths of FR groups compared with normal‐diet CON groups (Figure 1F). In addition, fat mobilization increased with increasing levels of FR. Notably, dietary changes significantly affect the size of body fat (Figure 1H). We found that adult mice in the FR15%–Re group had the highest levels of adiposity, even exceeding those in the HFD group, when we euthanized the remaining six mice per group at the end of 6 weeks of Re (Figure 1G). Hematoxylin and eosin (H&E) staining showed that FR15%–Re also had a significant effect on the histomorphology of white adipose tissue. In the quantitative plot, 1 represents the cell size of the control adipose tissue; the larger the cell, the higher the value (Figure 1I). In contrast, FR15%–Re in young mice had the lowest levels of fat mass and content during Re, even lower than normal‐diet CON groups (Figure S3). Oil Red O staining showed that FR15%–Re in the younger group also had a significant effect on the histomorphology of white adipose tissue (Figure S1D). In conclusion, the metabolic status of the mice was significantly affected by the FR15%–Re diet regardless of age, but not the FR40%–Re diet.

We later investigated the effects of two levels of FR on food intake in mice after resumption of ad libitum feeding. At the end of the 2‐week FR intervention, food intake in the two Re groups of adult mice spiked on the first day, followed by a sharp decline. Nevertheless, after 6 weeks of Re, there was no significant difference in food intake indexes between the two FR–Re groups (Figure 1D). The same trend was shown for the two restricted food groups in young mice (Figure S2). Thus, these results suggest that the abnormalities in lipid metabolism in FR15%–Re mice compared with FR40%–Re mice, irrespective of age, are not caused by the amount of food consumed ad libitum after Re.

### 2.2 FR15%–Re diets affected energy intake, digestibility, and tissue morphology regardless of age

**FIGURE 2:**
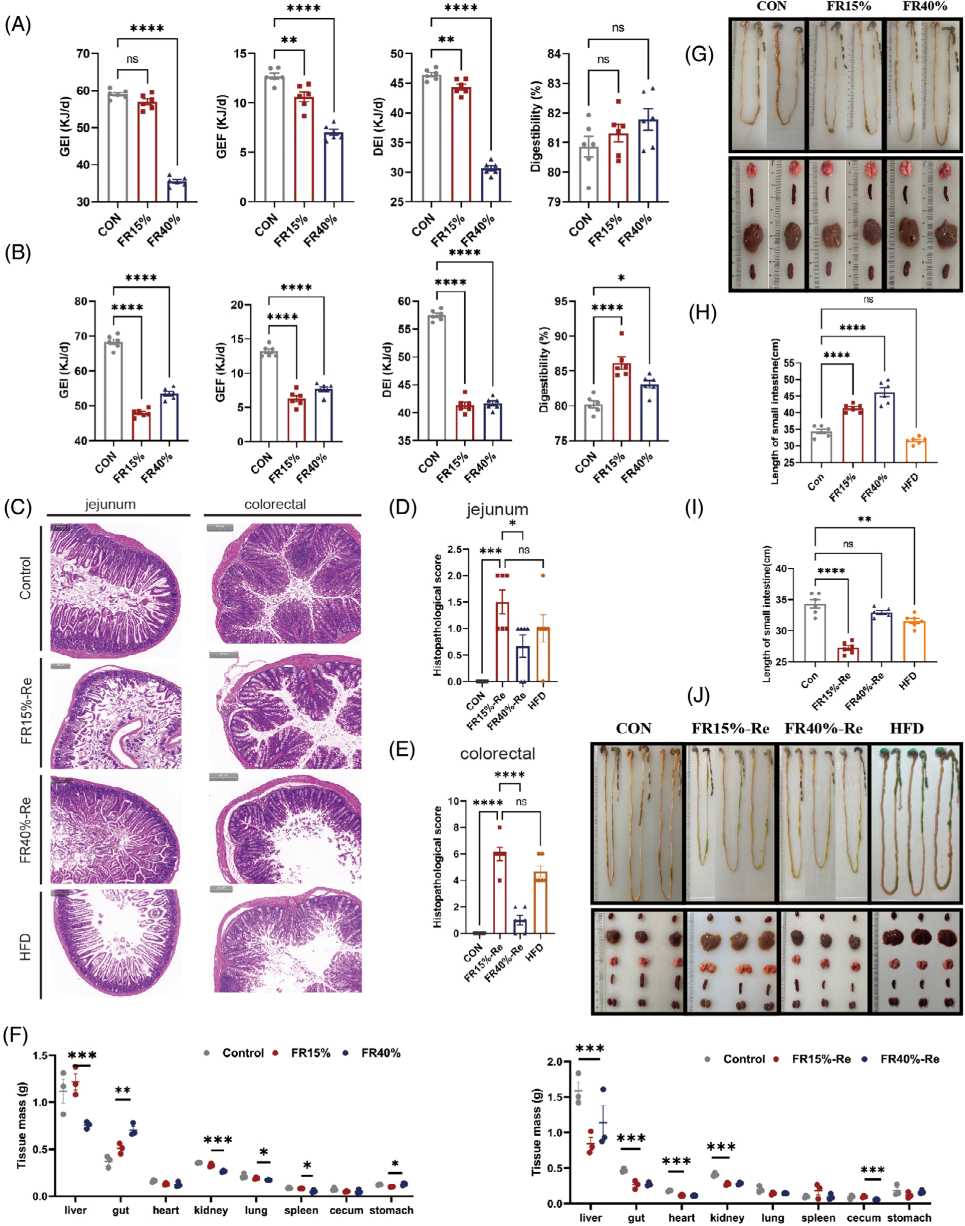
Observations on how food restriction and refeeding affect energy intake, digestibility, and tissue morphology. (A) Gross energy intake (GEI), gross energy of feces (GEF), digestive energy intake (DEI), and digestibility of the food‐restricted mice. (B) GEI, GEF, DEI, and digestibility of the food‐restricted mice refed with a high‐fat diet. (C) H&E‐stained paraffin slices of the jejunum and colorectal tissues from two FR–Re groups. Bars indicate 100 µm. (D) Histopathological scores of jejunum tissue sections (histological score = (inflammation + depth of lesions + crypt damage) × width of lesions). (E) Histopathological scores of colorectal tissue sections. (F) Tissue mass of food‐restricted mice and of food‐restricted mice fed a high‐fat diet. (G) Tissue morphology of food‐restricted mice. (H) Length of small intestine in food‐restricted mice (I) Length of small intestine in food‐restricted mice fed with high‐fat chow (J) Tissue morphology in food‐restricted mice fed with high‐fat chow. CON, the animals were fed ad libitum throughout the experiment; HFD, the animals were fed 60% high‐fat chow throughout the experiment; FR15% and FR40%, the animals were restricted by 15and 40% of ad libitum food intake for 2 weeks, and followed by high‐fat diet refeeding for 6 weeks (Re). Data were represented as mean ± SEM. *n* = 6 mice in each group. *p < 0.05, **p < 0.01, ***p < 0.001, ****p < 0.0001.

To assess the energy intake and digestibility of FR15%–Re and FR40%–Re diets, mice of different ages were restricted from eating for 2 weeks and then re‐fed a high‐fat diet for 6 weeks or more until significant differences occurred. Restriction of food intake was the most common cause of weight loss in animals.^25^, ^26^ We first focused on fecal energy in mice after 2 weeks of FR, and FR40% mice of different age groups consumed less food and had significantly lower digestive energy intake (DEI) compared with CONs (Figure 2A). The two FR groups of adult mice produced less total energy intake (gross energy intake [GEI]) and total fecal energy (gross energy of feces [GEF]), whereas FR did not affect digestibility (Figure 2A). In addition to digestibility, young mice treated with FR40% showed a similar significant trend of decreased GEI, GEF, and DEI (Figure S4).

Subsequently, we focused on fecal energy in mice of different ages 6 weeks after resumption of free access to food. Our results showed that adult male mice treated with FR15%–Re and FR40%–Re exhibited 21.3 and 10.3% lower GEI, respectively, and generated 64.3 and 54.6% fewer feces than CONs (Figure 2B). However, the DEI of the FR15%–Re group was 11.3% lower, but the digestibility was 11.4% higher than that of the CON group (Figure 2B). Furthermore, young mice treated with FR15%–Re processing had a similar tendency toward greater digestibility (Figure S5). These findings suggested that FR15%–Re diets have a significant effect on DEI and digestibility compared with FR40%–Re, regardless of age.

According to a previous study, metabolic diseases that influence lipid metabolism are marked by defective and compromised intestinal function.^24^ To examine the effect of FR–Re on intestinal tissue at two depths, we revealed histomorphology and inflammatory infiltration of the jejunum and colorectum by H&E staining and corresponding histological scoring. Our results showed that the jejunum and colorectal tissues of adult mice developed inflammatory lesions in the Re phase (Figure 2C). Among them, the histopathological scores of the FR15%–Re group were significantly different from those of the other groups (Figure 2D,E). And the FR15%–Re group had significantly lower tract mass (Figure 2F), and the length of the small and large intestines in the FR15%–Re group was also significantly shorter (Figure 2G–J). In addition, FR15%–Re also significantly damaged distal organs such as the liver compared with FR40%–Re (Figure S6).

### 2.3 FR15%–Re mice exhibited increased inflammatory markers

**FIGURE 3:**
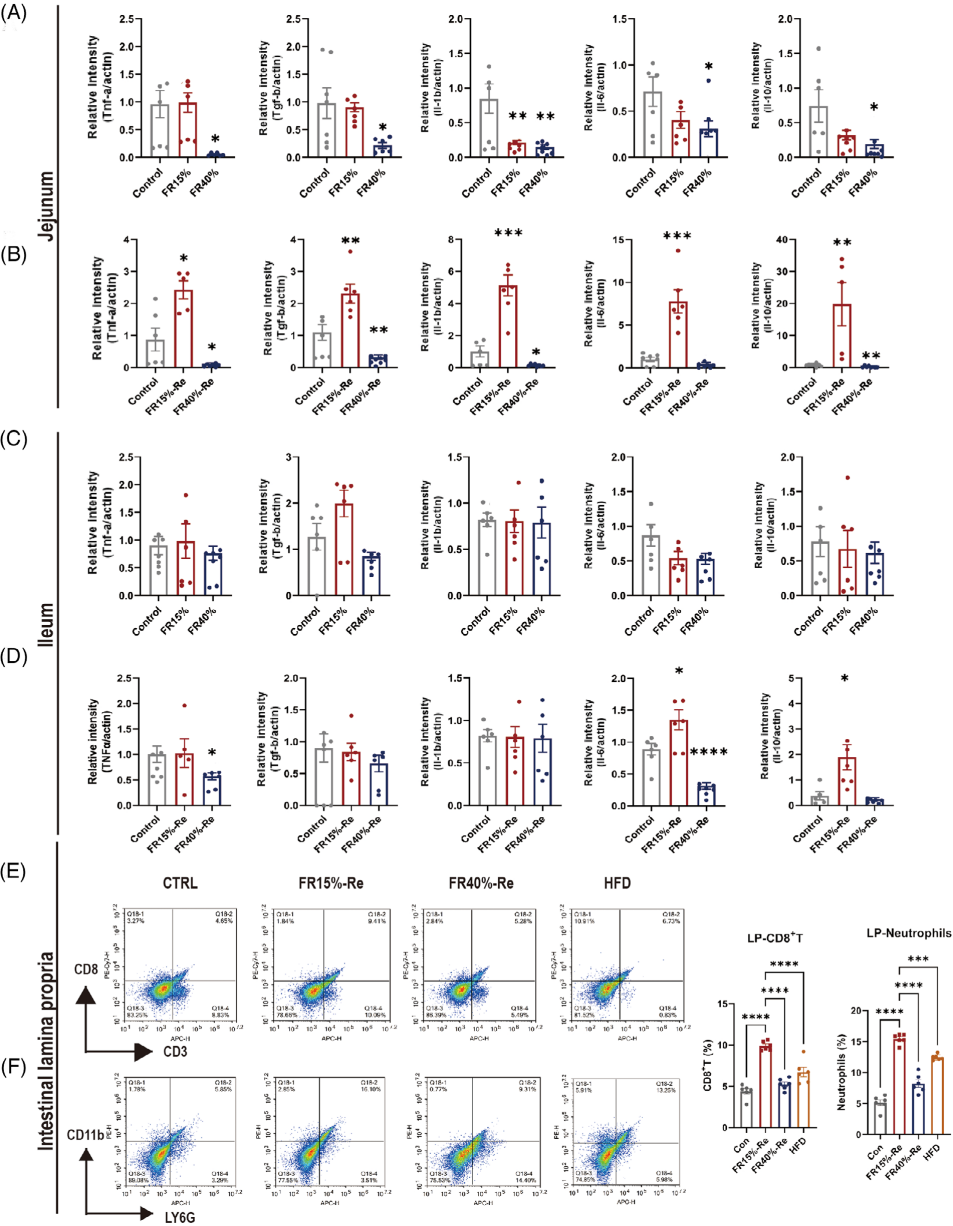
Inflammatory cytokines and immune cell subpopulations are produced during food restriction and refeeding. (A) Gene expression of Tnfa, Tgfb, Il‐1b, Il‐6, and Il‐10 in jejunum tissues was detected by quantitative real‐time PCR of the food‐restricted mice. (B) Gene expression of Tnfa, Tgfb, Il‐1b, Il‐6, and Il‐10 in jejunum tissues were detected by quantitative real‐time PCR of the food‐restricted mice refed with a high‐fat diet. (C) Gene expression of Tnfa, Tgfb, Il‐1b, Il‐6, and Il‐10 in ileum tissues was detected by quantitative real‐time PCR of the food‐restricted mice. (D) Gene expression of Tnfa, Tgfb, Il‐1b, Il‐6, and Il‐10 in ileum tissues were detected by quantitative real‐time PCR of the food‐restricted mice refed with a high‐fat diet. (E) CD8+T cells subpopulations in the intestinal tissue in the food‐restricted mice refed with a high‐fat diet. (F) Neutrophils cell subpopulations in the intestinal tissue in the food‐restricted mice refed with a high‐fat diet. CON, the animals were fed ad libitum throughout the experiment; HFD, the animals were fed 60% high‐fat chow throughout the experiment; FR15%–Re, FR40%–Re, the animals were restricted by 15 and 40% of ad libitum food intake, respectively, for 2 weeks and followed by high‐fat diet refeeding for 6 weeks. Data were represented as mean ± SEM. *n* = 6 mice in each group. *p < 0.05, **p < 0.01, ***p < 0.001, ****p < 0.0001.

It is known that increased intestinal permeability and consequent immune cell infiltration are thought to boost the generation of proinflammatory cytokines in immune and epithelial cells.^26^ To analyze the effects of the FR15%–Re group in more detail, we chose to analyze the adult mice with more pronounced and relevant changes in intestinal phenotype in further detail. As a result, we investigated inflammatory cytokine production in each intestinal segment after FR–Re treatment in adult mice through real‐time fluorescence quantitative polymerase chain reaction (qPCR). In the jejunum (Figure 3A,B) and ileum (Figure 3C,D), the production of inflammatory cytokines tumor necrosis factor‐a (Tnf‐a), interleukin (Il)‐1b, Il‐6, and immunomodulatory cytokines transforming growth factor‐b (Tgf‐b) and Il‐10 in FR15%–Re group was drastically increased (Figure 3B,D). Furthermore, these inflammatory cytokines remained high until the animals were terminated at day 60. This result suggests that mild FR15%–Re rather than severe FR40%–Re‐induced inflammation can persist for a long time, indicating the presence of chronic inflammation.

To better understand enteritis, we detected immune cell subpopulations in adult mice intestinal tissue at the appropriate moment (FR and Re). CD8^+^ (Cluster of differentiation 8^+^) T cells in the intestinal tissues were significantly higher in the FR15%–Re induced enteritis (9.41%) than in the CON group (4.65%) and HFD group (6.73%) (Figure 3E). In addition, the FR15%–Re groups had significantly more neutrophil cells (16.1%) in the intestinal tissues than the CON group (5.85%) and HFD group (13.25%) (Figure 3F). In general, these findings suggest that FR15%–Re animals maintain significant effects on fat accumulation, intestinal damage, and elevated inflammatory markers over time after switching from FR to Re compared with FR40%–Re mice, and even more so than the HFD group, which had been fed a high‐fat diet.

### 2.4 FR–Re mice showed different fecal microbiota changes from those of CON mice regardless of age

**FIGURE 4:**
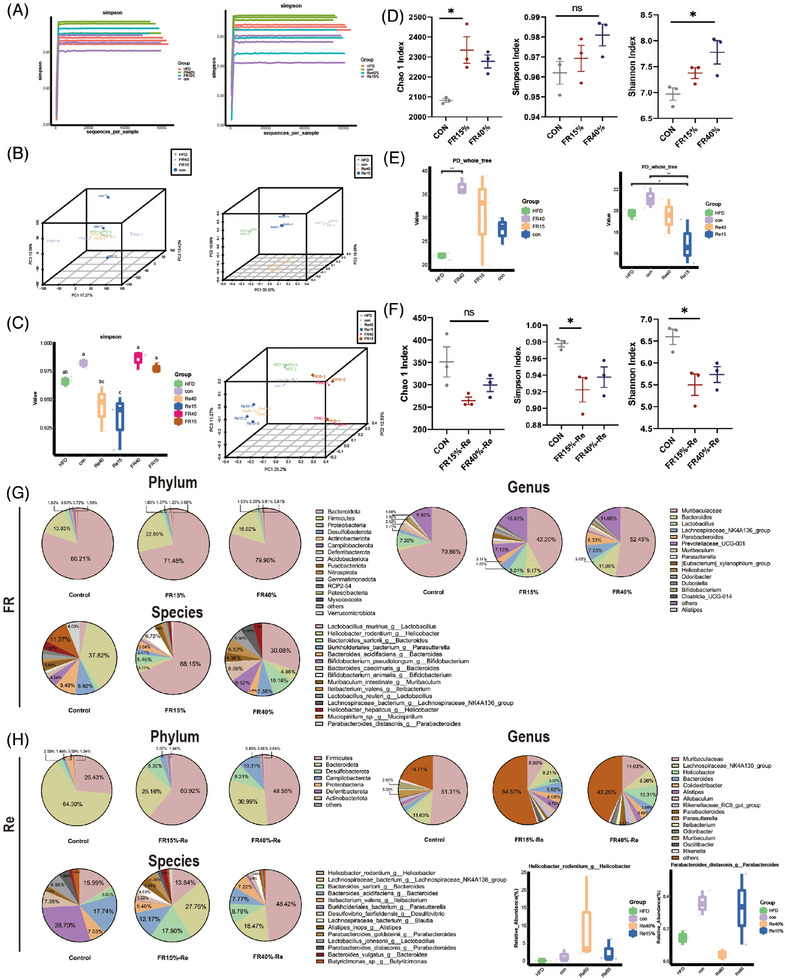
Food restriction and refeeding of mice alter the fecal microbiota. (A) Fecal samples from two FR–Re groups investigated the gut microbiota by sequencing the V3–V4 region of the 16S rRNA gene. (B) Two FR groups were subjected to principal coordinate analysis (PCoA). (C) Alpha diversity changed during the food‐restriction and refeeding period. (D) The Chao1 and Simpson index levels changed during the food‐restriction period. (E) Alpha diversity changed during the refeeding period. (F) The Chao1 and Simpson index levels changed during the refeeding period. (G) Phylum levels of microbiota composition in the two FR groups. Microbiota genus composition in the two FR groups. Microbiota species levels in the two FR groups. (H) Phylum levels of microbiota composition in the two FR–Re groups. Genus levels of microbiota composition in the two FR–Re groups. Microbiota species composition in the two FR–Re groups. CON, the animals were fed ad libitum throughout the experiment; HFD, the animals were fed 60% high‐fat chow throughout the experiment; FR15%–Re, FR40%–Re, the animals were restricted by 15 and 40% of ad libitum food intake, respectively, for 2 weeks and followed by high‐fat diet refeeding for 6 weeks. Data were represented as mean ± SEM. *n* = 6 mice in each group. *p < 0.05, **p < 0.01, ***p < 0.001, ****p < 0.0001.

Microbial oscillations are mainly driven by food consumption rhythmicity.^16^, ^27^ Therefore, we collected fecal samples from three groups at week 2 of FR and week 6 after switching to ad libitum feeding and investigated the gut microbiota by sequencing the V3–V4 region of the 16S rRNA gene (Figure 4A). To exclude confounding time factors, we compared the gut microbiota at the same time points. According to principal coordinate analysis (PCoA) and multivariate analysis based on Bray–Curtis distance (permutational multivariate analysis of variance), the gut microbiota structure of the adult mouse FR15% and FR40% mouse groups was significantly different from the CON mice after 2 weeks of FR (Figure 4B). FR and Re are the key factors that modify the gut microbiota, as shown by the first principal component (the first principal component, PC1), which is independent of the sample collecting individuals. Noteworthy, in the FR15%–Re group, gut microbiota structure changed dramatically, and the aggregate of gut microbiota isolated was markedly different from that of other groups (Figure 4B). The alterations in the gut microbiota were more pronounced in the FR15%–Re group, possibly because the mild restriction‐Re has a more pronounced effect on the organism. The PCoA plot and the results revealed that four groups in two periods had different gut microbiota structures (Figure 4B).

Further analysis showed that the adult FR–Re animals had considerably less alpha diversity during the Re phase than the CON mice (Figure 4C), which could be due to the higher food intake once the mice transitioned to ad libitum feeding. The gut microbiota of the FR–Re mice was significantly different from that of the CON mice according to the PCoA score plots (Figure 4B). Compared with the CON group, the mice in the FR groups showed a significant increase in the Chao1 index, which indicates species richness and the Simpson, Shannon index indicating diversity (Figure 4D). And, the FR15%–Re mice in the Re period showed significantly lower Chao1, Simpson, and Shannon indices than FR40%–Re and CON mice, which excluded dietary factors. Consistently, the gut microbiota alpha diversity of the FR15%–Re group was also much lower (Figure 4E,F). Furthermore, when compared with the CON group, the gut microbiota of the Re mice shifted to the right side of the PC1 axis, with the direction of change opposite that of the FR phase (Figure 4B). Furthermore, the young mice in the Re period showed a similar tendency of lower Chao1 and Simpson index (Figure S7A,B), and the gut microbiota shifted to the right side of the PC1 axis (Figure S7C). In conclusion, the gut microbiota of FR15%–Re mice was significantly different than that of FR40%–Re and CON mice, regardless of age.

In addition we found that mild FR–Re (FR15%–Re) affected fecal microbiota composition. Using sparse partial least squares discriminant analysis, members of the gut microbiota were identified as receiving FR–Re.^28^ To begin with, FR15% and FR40% mice were contrasted to comprehend the effect of FR levels on gut microbiota composition (Figure 4G). Especially in the FR15% group, Lactobacillus was considerably elevated in the FR mice, whereas FR15% mice exhibited a considerable inhibition of *Helicobacter* (Figure 4G). After different levels of FR–Re diet treatment, FR40%–Re animals had higher levels of *Helicobacter* (Figure 4H), whereas FR15%–Re mice had higher levels of enteritis‐related *Lachnospiraceae* and *Bacteroides. Alistipes* in the FR15%–Re group also displayed increased expression (Figure 4H). Additionally, observing and comparing microbial aggregation can be further done by using cluster maps of community features between various groupings (Figure S8). In the sequencing of FR15%–Re young mice, we likewise found a significant fecal microbiota composition change and a significant increase in *Lachnospiraceae* (Figure S9). These findings are consistent with our previous observation that host inflammation and unfavorable metabolic profiles may be closely associated with FR15%–Re‐induced changes in the associated intestinal microbiota.

### 2.5 Mild FR–Re impacts fecal microbiota composition

**FIGURE 5:**
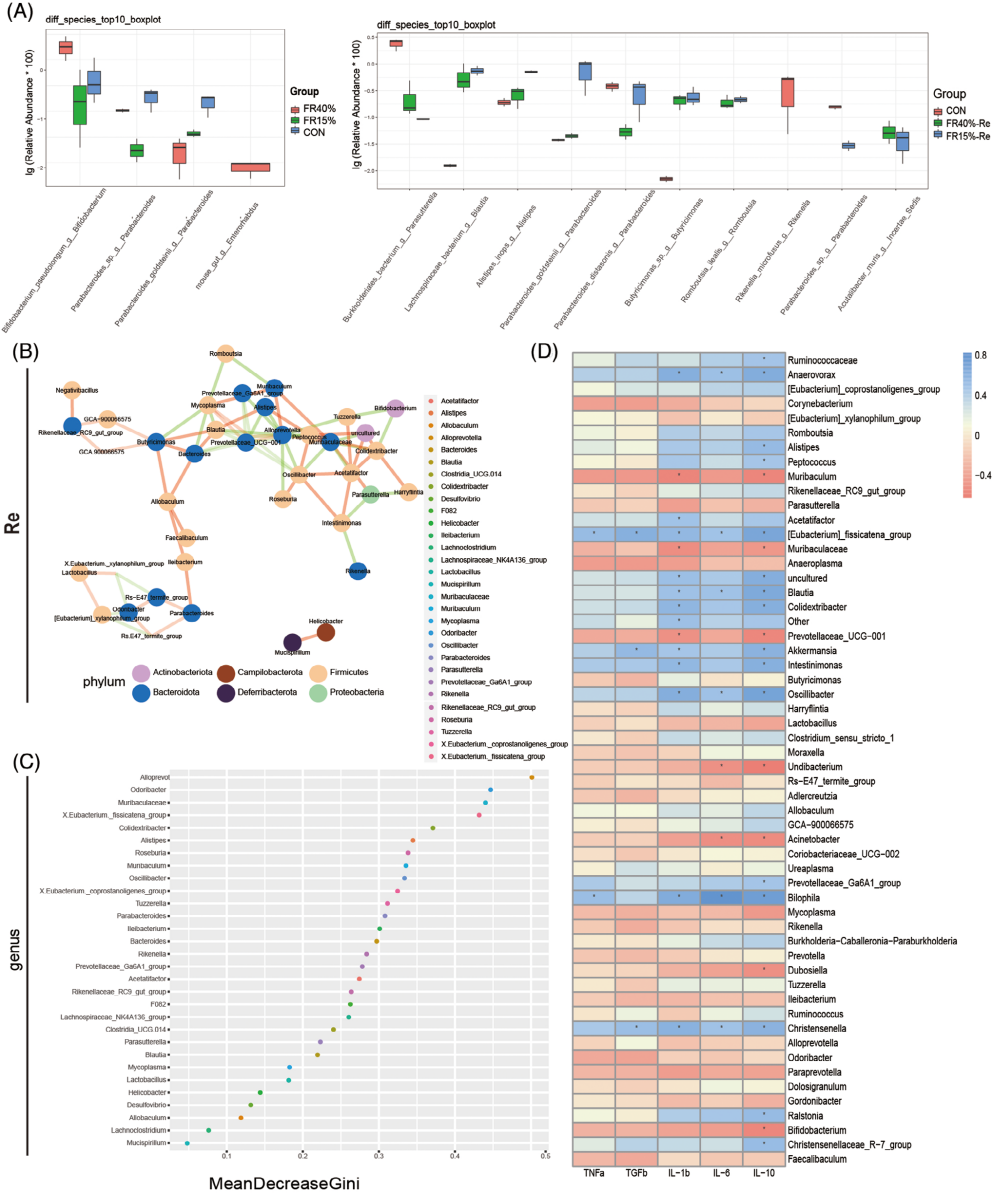
Food restriction and refeeding impact fecal microbiota composition. (A) Top 10 species‐level microbiota differences between the two FR groups. (B) The two FR–Re groups’ microbiota were analyzed for correlation. (C) Random forest analysis. (D) Association of microbiota with inflammatory factors. CON, the animals were fed ad libitum throughout the experiment; FR15%–Re, FR40%–Re, the animals were restricted by 15 and 40% of ad libitum food intake, respectively, for 2 weeks and followed by high‐fat diet refeeding for 6 weeks. Data were represented as mean ± SEM. *n* = 6 mice in each group. *p < 0.05, **p < 0.01, ***p < 0.001, ****p < 0.0001. Positive correlations are in red and negative correlations are in green.

Subsequently, when comparing species differences between the FR and Re groups in adult mice, we found that *Parabacteroides* was greatly reduced in the FR group (Figure 5A), but significantly increased in the Re stage (Figure 5A). In addition, *Lachnospiraceae* and *Alistipes* were much more abundant in the FR15%–Re group than in the other groups, which was noteworthy (Figure 5A). In addition, differences in the top 10 species‐level microbiota between CON and FR15% mice were significantly higher for microbiota such as *Alistipes* and *Lachnospiraceae* after mild FR and Re (Figure S10). Consistent with this, the younger FR15%–Re group also showed higher levels of variation in *Lachnospiraceae* and *Bacteroidota* isotypes (Figure S11). These results imply that FR and Re can each be enhanced for specific microbiota. Furthermore, the bacterial microbiota was drastically altered by FR15%–Re, which may be strongly related to the adverse phenotype(Figures 5D and S12).

Further microbiota correlation analysis revealed that the highly expressed *Bacteroides_g* in the FR15%–Re group belonged to *Bacteroidota_p*, which was significantly enriched in the FR–Re group and positively connected with *Alistipes_g*, which belonged to the same phylum (Figure 5B). The group of *Parabacteroides_g* (Figure 5A) showed a favorable correlation as well. It is interesting to note that *Firmicutes_p*, a member of the *Lachnospiraceae_g*, showed a positive correlation with *Bacteroidota_p* (Figures 5B,C and S13). This is in line with the preceding traits of community composition changes. Overall, the positive correlations between the FR15%–Re‐enriched microbiota and enteritis suggested that the microbiota may work in concert to affect the metabolic profile and inflammation of the host (Figure 5D).

### 2.6 Fecal transplantation improves phenotype and related cytokine expression in FR15%–Re mice regardless of age

To determine whether FR15%–Re‐mediated interactions between lipid metabolism, gut microbiota, and inflammatory factors could be mitigated, we performed a fecal microbiota transplantation (FMT) experiment. After 1 week of mouse acclimatization, mice of both ages were randomly divided into a total of eight groups, and donor mice were divided into four groups: (1) adult CON group (*n* = 6): mice were free to consume food and water for 2 months; (2) young CON group (*n* = 6): mice were free to consume food and water for 2 months. (3) adult FR15%–Re group (*n* = 6): adult mice were fed and watered ad libitum for 7 days, and reintroduced to high‐fat ad libitum for 40 days after 14 days of 15% diet restriction; (4) young FR15%–Re group (*n* = 6): young mice were fed and watered ad libitum for 7 days, and reintroduced to high‐fat ad libitum for 40 days after 14 days of 15% diet restriction. At 2 months of modeling, fresh fecal samples were collected daily and transplanted into FMT‐recipient mice. Recipient mice were similarly divided into four groups, where two groups of FMT‐recipient mice experiencing FR15%–Re were transplanted with fecal samples from CON donor mice (Con‐FMT), and two groups of age‐differentiated CON FMT‐recipient mice were transplanted with fecal samples from donor mice in the FR15%–Re group (FR15%–Re‐FMT).

**FIGURE 6:**
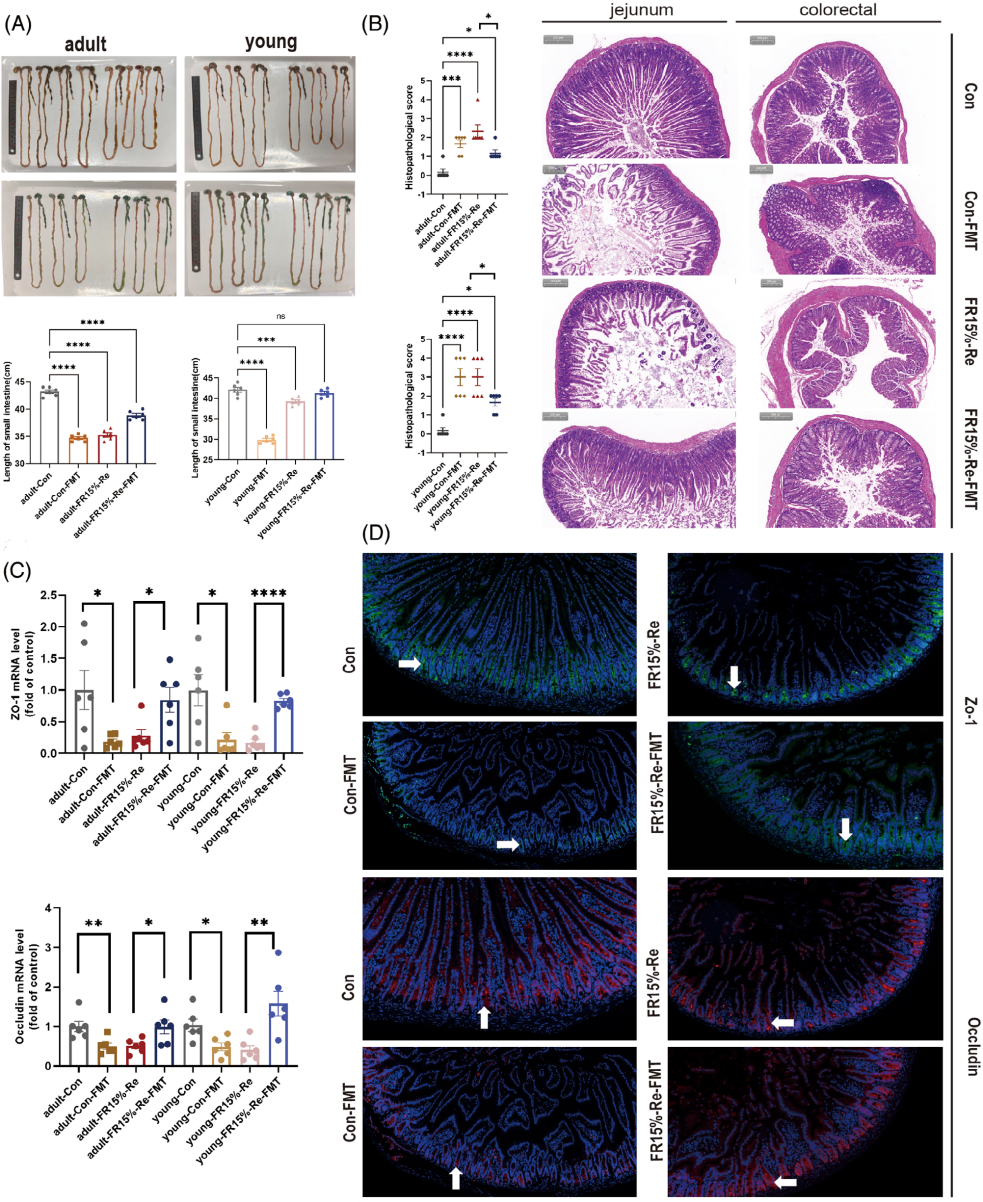
Fecal transplantation improves phenotype and related cytokine expression in FR15%–Re mice. (A) Intestinal length and its phenotype in eight groups of mice. (B) HE staining and pathological scoring of jejunum and colon in four groups of mice. (C) qPCR detection of intestinal permeability‐related gene expression in eight groups of mice. (D) Immunofluorescence double‐staining detection of intestinal permeability‐related protein expression. (1) adult CON group (*n* = 6): mice were fed and watered ad libitum for 2 months; (2) young CON group (*n* = 6): mice were fed and watered ad libitum for 2 months. (3) adult FR15%–Re group (*n* = 6): adult mice were fed and watered ad libitum for 7 days, and refed to high‐fat ad libitum for 40 days after 14 days of 15% diet restriction; (4) young FR15%–Re group (*n* = 6): young mice were fed and watered ad libitum for 7 days, and refed to high‐fat ad libitum for 40 days after 14 days of 15% diet restriction. At 2 months of modeling, fresh fecal samples were collected daily and transplanted into fecal microbiota transplant (FMT)‐recipient mice. Recipient mice were similarly divided into four groups, where two groups of FMT‐recipient mice experiencing FR15%–Re were transplanted with fecal samples from CON donor mice (Con‐FMT), and two groups of age‐differentiated CON FMT‐recipient mice were transplanted with fecal samples from donor mice in the FR15%–Re group (FR15%–Re‐FMT). Data were represented as mean ± SEM. *n* = 6 mice in each group.

Our results showed that the small intestine length of mice in the FR15%–Re‐FMT group was increased compared with FR15%–Re in both age groups (Figure 6A), that is, backfeeding CON feces phenotypically restored some of the intestinal breaks. mice in the Con‐FMT group had significantly shorter small intestine lengths compared with the Con group (Figure 6A), that is, feeding FR15%–Re feces similarly affected normal mice's intestinal phenotype of normal mice. Further HE staining results showed that the mice in the FR15%–Re group had the most severe intestinal damage and the highest score. This was followed by mice in the Con‐FMT group, whereas FR15%–Re‐FMT mice had improved intestinal damage scores (Figure 6B). Taken together, our results suggest that fecal transplantation restored intestinal inflammation and intestinal injury phenotype in mice in the FR15%–Re group, whereas the degree of intestinal injury was increased in Con‐FMT mice receiving FR15%–Re feces.

**FIGURE 7:**
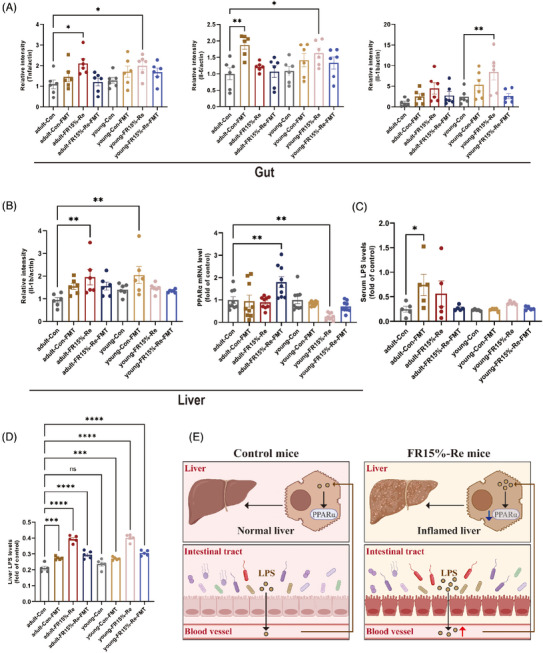
Association between FR15%–Re dietary influences on host lipid metabolism and inflammatory response and gut microbiota. (A) Expression of intestinal inflammatory factors in eight groups of mice. (B) Expression of hepatic inflammatory factors and PPAR‐α in eight groups of mice. (C) Serum LPS levels detected by ELISA in eight groups of mice. (D) Liver LPS levels detected by ELISA in eight groups of mice (E) Possible regulatory mechanisms. (1) Adult CON group (*n* = 6): mice were fed and watered ad libitum for 2 months; (2) young CON group (*n* = 6): mice were fed and watered ad libitum for 2 months. (3) Adult FR15%–Re group (*n* = 6): adult mice were fed and watered ad libitum for 7 days, and refed to high‐fat ad libitum for 40 days after 14 days of 15% diet restriction; (4) young FR15%–Re group (*n* = 6): young mice were fed and watered ad libitum for 7 days, and refed to high‐fat ad libitum for 40 days after 14 days of 15% diet restriction. At 2 months of modeling, fresh fecal samples were collected daily and transplanted into fecal microbiota transplant (FMT)‐recipient mice. Recipient mice were similarly divided into four groups, where two groups of FMT‐recipient mice experiencing FR15%–Re were transplanted with fecal samples from CON donor mice (Con‐FMT), and two groups of age‐differentiated CON FMT‐recipient mice were transplanted with fecal samples from donor mice in the FR15%–Re group (FR15%–Re‐FMT). Data were represented as mean ± SEM. *n* = 6 mice in each group.

To verify the modulatory effect of fecal transplantation on inflammatory factors in intestinal tissues, we assessed whether fecal transplantation treatment affects the production of proinflammatory cytokines. As expected, mRNA expression levels of proinflammatory cytokines Tnf‐a, Il‐1b, and Il‐6 were significantly reduced in FR15%–Re‐FMT mice of both ages (Figure 7A). In addition, we observed Con‐FMT mice of different ages and found that the mRNA expression levels of proinflammatory cytokines Tnf‐a, Il‐1b, and Il‐6 were significantly increased (Figure 7A). That is, regardless of age, fecal transplantation has a regulatory effect on inflammatory factors in intestinal tissues.

Further to verify the effect of fecal transplantation on intestinal epithelial barrier function, the tight junction protein ZO‐1 was considered, occludin is an indicator to assess the degree of intestinal inflammation and intestinal permeability in patients with enterocolitis, we performed qPCR experiments. As shown in Figure 6C, the expression levels of ZO‐1 and occludin were significantly decreased in the FR15%–Re group, while the FR15%–Re‐FMT group effectively increased the expression levels of both genes. To further explore the correlation, we performed immunofluorescence homology double labeling experiments. Compared with the normal CON group, ZO‐1 and occludin expression levels were significantly reduced in the Con‐FMT group, which was alleviated in the FR15%–Re‐FMT group compared with the FR15%–Re group, that is, the expression levels of the two proteins were significantly increased (Figure 6D). Consistent with the results of the previous animal experiments, fecal transplantation was able to improve the host intestinal phenotype induced by FR15%–Re.

### 2.7 A preliminary investigation of the link between FR15%–Re dietary influences on host lipid metabolism and inflammatory response and intestinal microbiota

The liver is the most important organ for systemic lipid metabolism and is regulated by transcription factors in the process of providing energy to the organism, among which PPARα is a core transcription factor. PPARα is mainly expressed in the liver and is mainly involved in β‐oxidation and fatty acid transport to regulate lipid homeostasis. To investigate the effects of FR15%–Re diet on the liver, we measured hepatic inflammatory factors and PPAR‐α. The results showed that hepatic Il‐1b was significantly increased in mice in the FR15%–Re group, which was alleviated by FMT treatment (Figure 7B), that is, high‐fat Re after a moderate dietary restriction has the potential to cause the development of liver disease.

In recent years, a large number of studies have shown that alterations in the intestinal microbiota as well as the endocytotoxin LPS produced by intestinal bacteria play an important role in the development of liver disease, especially hepatitis and fatty liver progression. In order to investigate whether LPS produced in the intestine affects the liver through the intestinal barrier, we performed enzyme‐linked immunosorbent assay (ELISA) experiments to determine the levels of LPS in serum and liver, respectively. The experimental results showed that the LPS content in the liver was significantly higher in the FR15%–Re group compared with the CON group (Figure 7C), whereas the LPS content in the serum was also highly significantly higher in the FR15%–Re group at both ages (Figure 7D); furthermore, the LPS content in the serum and liver was downregulated in the FR15%–Re‐FMT group (Figure 7C,D). In addition, the serum and liver LPS levels were significantly higher in the Con‐FMT group at both ages, that is, indicating that the feces of mice in the FR15%–Re group also impaired the phenotype and metabolism of mice in the Con group, among others.

In summary, we learned that the intestinal barrier is impaired by immunofluorescence homology double labeling experiments, that is, LPS can enter the serum through the intestine. After further serum and liver LPS measurements, we preliminarily determined that the FR15%–Re dietary strategy may induce the LPS produced by the intestinal microbiota to pass through the intestinal barrier and thus enter the liver through the serum, thus inducing the development of liver disease (Figure 7E). In addition, the liver is an important organ involved in lipid metabolism, and we found that the expression of PPAR‐α, which is mainly expressed in the liver, was altered, with a significant decrease in the FR15%–Re group, and a rebound improvement after receiving FMT (Figure 7B), which was negatively correlated with the amount of LPS produced and delivered by the intestinal tract. The literature search further validated that PPAR‐α reduces inflammation and reverses LPS production. Accordingly, we tentatively hypothesized that the link between the R15%–Re diet affecting host lipid metabolism and inflammatory response and intestinal microbiota may be closely related to the intestinal–hepatic regulation by PPAR‐α and LPS (Figure 7E).

## 3 DISCUSSION

According to our findings, the degree of FR affects the effects of Re induction, including physiological alterations, gut microbiota modulation, and proinflammatory changes, all of which could be harmful to health. The FR–Re protocol has two main components: (i) limited food availability for a set length of time in the absence of malnutrition; and (ii) Re feeding settings. Feeding parameters have a significantly differential impact on host organism health.^20^ Regrettably, the degree of FR and the consequences of Re are not described in most studies.^3^, ^29^ Current research suggests that the degree of FR is important and determines the organism's response to Re.

As many studies have reported, FR tends to have more positive results for weight loss.^1^, ^11^, ^21^ Consistent with this, our results showed that FR40% mice weighed much less than FR15% mice after 2 weeks of FR. The difference in body weight and intestinal tissue between the two FR–Re circumstances between the two groups of mice supports the notion that the level of restriction is significant. Previous studies have shown that fat deposition is more susceptible to lower degrees of restriction, leading to significant changes in the expression of genes associated with lipid metabolism after Re.^30^ This implies that restricting one's intake of food may have a beneficial effect, but can have a rebound effect depending on the degree of restriction. Interestingly, the FR40%–Re did not have more harmful and long‐lasting effects than the FR15%–Re.

Through its possible anti‐inflammatory effects, FR can significantly improve metabolic phenotypes.^20^ Consistently, our experimental results showed a considerable drop in inflammatory markers in FR mice. Following further Re, inflammatory markers were significantly upregulated in the intestinal tissues of FR15%–Re mice. Recent studies on the anti‐inflammatory benefits of FR found that FR‐induced changes in microbiota CON the tone of the immune response, boosting improvement by reducing endotoxin in mice,^5^ and establishing the gut microbiota of *L. murinus* as a determinant of positive outcomes.^6^ However, no studies have revealed which member of the gut microbiota is responsible for the proinflammatory effects of Re. Our results showed that FR15%–Re significantly altered the structure and content of the mouse fecal microbiota, with higher numbers of *Lachnospira* and *Bacteroides_sartorii* than FR40%–Re and CONs during Re. Currently, the most prevalent Lactobacillus OTU enhanced by FR has been identified from a strain of L. murinus^5^ and has been demonstrated to preserve the gut barrier and reduce inflammation.^9^ Therefore, the degree of inhibition of this strain by Re also could be linked to proinflammatory and metabolic consequences.

The occurrence of enteritis‐related diseases has been demonstrated to be closely correlated with increased gut microbiota members such as *Lachnospiraceae* and *Bacteroidetes*. In this study, the FR15%–Re group showed much higher expression of *Lachnospiraceae_bacterrium* and *Bacteroidetes_sartorii*. Gastric cancer can be caused by *Helicobacter* infection,^31^ and the Re group had the highest *Helicobacter*. Furthermore, representatives of the genus *Alistipes* have been linked to pain in patients with irritable bowel syndrome.^29^ This bacterium belongs to the same phylum as *Bacteroides*, which also displayed higher expression in the FR15%–Re groups. In order to understand whether the phenotypic and metabolic changes caused by the FR15%–Re diet are plastic, we found that FMT could alleviate the improvement of the situation through fecal transplantation experiments, and we also found that the Con‐FMT group receiving fecal transplants from the FR15%–Re group showed the same negative phenotypic damage to the liver and intestines as well as the recruitment of inflammatory factors.

In recent years, a large number of studies have shown that the alteration of intestinal microbiota as well as the cytotoxin LPS produced by intestinal bacteria play an important role in the development of liver disease, especially hepatitis and fatty liver progression. Although the liver is the most important organ for systemic lipid metabolism, it is regulated by transcription factors in providing energy to the body, among which PPAR‐α is a central transcription factor. To investigate whether LPS produced in the intestine affects the liver through the intestinal barrier, we first determined that the expression of intestinal permeability proteins ZO‐1, occludin, was downregulated in the FR15%–Re group, that is, intestinal permeability was impaired by qPCR and fluorescence double‐staining experiments. Subsequently, ELISA experiments were performed to determine the LPS content in serum and liver, respectively, and it was found that LPS was significantly increased in serum and upregulated in liver in the FR15%–Re group. This initially verified that LPS passed through the damaged intestinal barrier and affected the liver through serum. We further determined the expression of PPAR‐α in the liver and found that its expression was negatively correlated with LPS. Accordingly, we preliminarily hypothesized that the link between the R15%–Re diet affecting host lipid metabolism and inflammatory response and intestinal microbiota may be closely related to the intestinal–hepatic regulation by PPAR‐α and LPS.

Studies have shown that FR15% and FR40% reduce body weight and adiposity, and this result has also been reported in clinical trials, that is, the phenomenon is consistent in humans and mice.^40−43^ The results of FR at different ages suggest that Re after moderate FR can have long‐lasting negative effects, including more significant changes in host body weight, fat accumulation, energy metabolism, and immune cell recruitment. When FR ends, less weight loss may result in abnormal body fat accumulation, liver damage, and intestinal inflammatory factors, and immune cell recruitment, which may involve several types of altered intestinal microbiota. Further fecal transplantation experiments and serum and liver ELISA experiments preliminarily suggested that the link between lipid metabolism and inflammatory response and intestinal microbiota might be related to PPAR‐α and LPS regulation of the gut‐hepatic interface. In conclusion, by comparing the fecal microbiota of the FR and Re groups, we found that the FR15% group produced more Lactobacillus while Helicobacter was significantly suppressed. Throughout the Re phase, Helicobacter was significantly enriched in the FR–Re group. As a result of obesity, there will be a greater focus on diet and dietary restrictions^22^ and our results focused on the recovery after two depths of FR. Our results emphasize the altered metabolic phenotype and gut microbiota and suggest that FR15%–Re may be involved in altered gut microbiota, more deleterious metabolic phenotype and inflammation. And that such results are potentially reversible by fecal transplantation. Currently, the interesting phenomenon of animal experiments including the ameliorative effect of fecal transplantation on FR15%–Re has not yet been clinically reported, so conducting similar clinical trials would be desirable. We look forward to more in‐depth mechanistic studies to help us understand the factors that contribute to enteritis and metabolic phenotypes of Re syndrome as well as the influence of gut microorganisms, which will help us to formulate more precise preventive and therapeutic strategies.

## 4 MATERIALS AND METHODS

### 4.1 Ethical review

All animal experiment procedures were approved by the Nanjing University Animal Care and Use Committee. This study was not designed with death as an endpoint, but rather particular dietary interventions were terminated when animals died unexpectedly.

### 4.2 Animal trial

The mice were purchased from Beijing Animal Centre (Beijing, China), and the experiments were performed in Individually Ventilated Cages (39 cm × 18 cm × 18 cm) with sawdust bedding. All mice were kept on a standard 12‐h light/dark cycle (lights on at 9:00 a.m.) and a temperature of 21 ± 3°C. All animals received humane care according to Chinese legal requirements.

#### 4.2.1 CON and HFD groups

The CON group fed adult (12 weeks old, *n* = 12) and young (6 weeks old, *n* = 12) C57BL/6J male mice a regular chow diet (standard rodent chow; 10% fat, 70% carbohydrate, 20% protein; calorific value 18.2 kJ/g) throughout the experiment. Different age HFD groups (*n* = 24) were fed high‐fat chow (XTHF60; 60% fat, 20% carbohydrate, 20% protein; calorific value 25.5 kJ/g) throughout. Mice were allowed to drink and eat freely. Mouse body weight and food intake per cage (to the nearest 0.1 g) were measured every 1−2 days.

#### 4.2.2 FR15% and FR15%–Re groups

Baseline measurements of food intake and body weight of adult (*n* = 12) and young mice (*n* = 12) were performed for a period of 1–2 weeks (days 1−13). After baseline measurements, mice (*n* = 12) were randomly divided into two groups of six mice each: (a) FR15% group (*n* = 6): housed for 2 weeks and restricted to 15% ad libitum food intake; and (b) FR15%–Re group (*n* = 6): housed for 6−10 weeks and fed ad libitum with a high‐fat diet (fat content of 60%, calorific value of 25.5 kJ/g; Chinese Research Diet). Both FR and Re mice were allowed to drink water ad libitum. Mouse body weights and food intake per cage (to the nearest 0.1 g) were measured every 1−2 days.

#### 4.2.3 FR40% and FR40%–Re groups

Food intake and body weight of young (*n* = 12) and adult (*n* = 12) mice were measured at baseline over a 2‐week period (days 1−13). After 2 weeks of feeding, mice (*n* = 12) were randomly divided into two groups of six mice each: (a) FR40% (*n* = 6): fed for 2 weeks with 40% restriction of ad libitum food intake; and (b) FR40%–Re (*n* = 6): fed for 6−10 weeks with a high‐fat diet (60% fat content, calorific value of 25.5 kJ/g; the Chinese Research Diet). FR and Re mice were allowed to drink water ad libitum. Mouse body weights and food intake per cage (to the nearest 0.1 g) were measured every 1−2 days.

### 4.3 Energy intake and digestibility

GEI and digestibility were measured between the last 2 days of FR and Re, using a food balance method described previously. Food was provided quantitatively over the last 2 days, and uneaten food and feces were collected 48 h later. The spillage of food and feces was separated manually after drying at 60°C for 10 days to constant mass. The gross energy contents of the food and feces were determined using a bomb calorimeter (C2000). GEI, gross energy of feces (GEF), DEI, and digestibility were calculated based on the following equations^32^:

$$\begin{array}{ccc} & & \mathrm{GEI}\left(\mathrm{kJ}/d\right)\\ & = & [\mathrm{food}\;\mathrm{provided}\;\left(g/\;d\right)\times \mathrm{dry}\;\mathrm{matter}\;\mathrm{content}\;\mathrm{of}\;\mathrm{food}\\ & & \left(\%\right)\;-\mathrm{dry}\;\mathrm{spillage}\;\mathrm{of}\;\mathrm{food}\;\mathrm{and}\;\mathrm{uneaten}\;\mathrm{food}]\\ & & \times \mathrm{gross}\;\mathrm{energy}\;\mathrm{content}\;\mathrm{of}\;\mathrm{food}\;\left(\mathrm{kJ}/g\right);\\ & & \mathrm{GEF}\left(\mathrm{kJ}/d\right)=\mathrm{dry}\;\mathrm{feces}\;\mathrm{mass}\;\left(g/d\right)\\ & & \times \mathrm{gross}\;\mathrm{energy}\;\mathrm{content}\;\mathrm{of}\;\mathrm{feces}\left(\mathrm{kJ}/g\right);\\ & & \mathrm{DEI}\left(\mathrm{kJ}/d\right)=\mathrm{GEI}-\mathrm{GEF};\;\mathrm{and}\;\mathrm{digestibility}\left(\%\right)\\ & = & \mathrm{DEI}/\mathrm{GEI}\times 100\%. \end{array}$$

### 4.4 Body composition

Fresh feces collection was performed prior to animal sampling, and fresh feces excreted by mice in a sterile environment were collected in sterilized centrifuge tubes every morning for 3 days after successful modeling and quickly snap‐frozen in liquid nitrogen. Afterward, the animals were euthanized with a RWD anesthesia ventilator (Catalog NO: R540‐48), and then blood was collected. After blood collection, hypothalamus, subcutaneous fat, and liver were carefully and quickly separated. All tissues were frozen in liquid nitrogen and stored at −80°C until analysis. Stomach, small intestine, large intestine, cecum, liver, heart, lung, spleen, and kidney were separated and weighed (to 1 mg) after separation. These tissues and the remaining carcass were weighed to determine the wet mass (to 1 mg). The carcass was dried in an oven at 60°C to a constant mass for 2 weeks, and then reweighed (to 1 mg) to determine its dry mass.^33^

### 4.5 Immunofluorescence homology double labeling assay on paraffin sections

The center part of each intestine was preserved in paraformaldehyde for 24 h. Then after paraffin section deparaffinization to water, antigen repair, drawing circle serum closure, addition of primary antibody and HRP secondary antibody, fluorescent dye reaction, antibody elution, DAPI restaining of cell nuclei after repeating the steps of the second round of labeling, quenching tissue autofluorescence, and sealing of the film; and finally microscopic examination and photographing.

### 4.6 Oil red O fat staining

Red O is a fat‐soluble dye that is highly soluble within fat and can specifically color neutral fats such as triglycerides in tissues. First, the sections were dried and slightly washed in 50% ethanol; oil red O ethanol dye solution acted for 8 min; 50% ethanol differentiation, tap water terminated differentiation; hematoxylin restaining nuclei, tap water returned to the blue, glycerol gelatin sealing. The fat droplets in the hepatocytes were red and the nuclei were blue.

### 4.7 Fecal microbiota analysis by 16s rRNA sequencing

#### 4.7.1 DNA extraction and amplification

After collection, cecal content samples were snap frozen and stored at −80°C. In this study, the actual number of mice used in each group was six. Before sequencing, we mixed stool samples from two mice in each group. Bacterial DNA was isolated from the cecal contents using a MagPure Soil DNA LQ Kit (Magen, Guangdong, China) following the manufacturer's instructions. DNA concentration and integrity were measured by a NanoDrop 2000 spectrophotometer (Thermo Fisher Scientific, Waltham, MA, USA) and agarose gel electrophoresis, respectively. PCR amplification of the V3–V4 hypervariable regions of the bacterial 16S rRNA gene was carried out in a 25 µL reaction using universal primer pairs (343F: 5′‐TACGGRAGGCAGCAG‐3′; 798R: 5′‐AGGGTATCTAATCCT‐3′). The reverse primer contained a sample barcode, and both primers were connected with an Illumina sequencing adapter.

#### 4.7.2 Library construction and sequencing

The Amplicon quality was visualized using gel electrophoresis. The PCR products were purified with Agencourt AMPure XP beads (Beckman Coulter Co., USA) and quantified using the Qubit dsDNA assay kit. The concentrations were then adjusted for sequencing. Sequencing was performed on an Illumina NovaSeq6000 with two paired‐end read cycles of 250 bases each (Illumina Inc., San Diego, CA; OE Biotech Company, Shanghai, China).

#### 4.7.3 Bioinformatic analysis

Raw sequencing data are in FASTQ format. Paired‐end reads were then preprocessed using cutadapt software to detect and cut off the adapter. After trimming, paired‐end reads were filtered for low‐quality sequences, denoised, merged, and detected and cut off the chimera reads using DADA2 with the default parameters of QIIME2. Finally, the software outputs the representative reads and the ASV abundance table. The representative read of each ASV was selected using the QIIME 2 package. All representative reads were annotated and blasted against Silva database Version 138 (or Unite) (16s/18s/ITS rDNA) using q2‐feature‐classifier with the default parameters. The microbial diversity in cecal content samples was estimated using the alpha diversity that includes the Chao1 index and Shannon index. The Unifrac distance matrix performed by QIIME2 software was used for unweighted Unifrac PCoA and phylogenetic tree construction.

### 4.8 Fecal microbiota transplantation

After 1 week of mouse acclimatization, mice of both ages were randomized into a total of eight groups, and donor mice were divided into four groups: (1) adult CON group (*n* = 6): mice were fed and watered ad libitum for 2 months; (2) young CON group (*n* = 6): mice were fed and watered ad libitum for 2 months. (3) adult FR15%–Re group (*n* = 6): adult mice were fed and watered ad libitum for 7 days, and refed to high‐fat ad libitum for 40 days after 14 days of 15% diet restriction; (4) young FR15%–Re group (*n* = 6): young mice were fed and watered ad libitum for 7 days, and refed to high‐fat ad libitum for 40 days after 14 days of 15% diet restriction. At 2 months of modeling, fresh fecal samples were collected daily and transplanted into FMT‐recipient mice. Recipient mice were similarly divided into four groups, where two groups of FMT‐recipient mice experiencing FR15%–Re were transplanted with fecal samples from CON donor mice (Con‐FMT), and two groups of age‐differentiated CON FMT‐recipient mice were transplanted with fecal samples from donor mice in the FR15%–Re group (FR15%–Re‐FMT). For fecal transplantation, fresh feces from each group were pooled, homogenized, and diluted with sterile saline to a final concentration of 1 mg feces/10 µL. Pooled samples were centrifuged at 1006.2g for 5 min. The supernatant was collected through a 70 µm filter and each mouse was gavaged (10 mL/kg) for 5 consecutive days.

### 4.9 Real‐time qPCR

Total RNA was extracted from jejunum and ileum samples using a TRIzol reagent (Invitrogen, Carlsbad, CA, USA). cDNA was synthesized with random primer oligo (dT) 18 and AMV reverse transcriptase (TAKARA) with a final reaction volume of 20 µL. A 2 µL cDNA sample was taken and subsequent PCR reaction was performed with gene‐specific primers (Table S1). The final reaction volume was 20 µL, containing 2× SYBR Premix EX Tag TM 10 µL, forward and reverse primers 0.4 µL (final concentration of 0.2 M for each primer), cDNA template 2 µL, and DEPC 7.2 µL. A quantitative real‐time PCR was performed using SsoFast EvaGreen Supermix on a CFX96 Real‐Time System (Bio‐Rad Laboratories, Hercules, CA, USA). Actin was used as an internal standard. Samples were quantified for the relative quantity of gene expression.^35^ The primers used in the quantitative real‐time PCR are listed in Table S1.

### 4.10 Flow cytometry

The intestinal tissue was cut into 0.5 cm pieces and kept in complete medium (RPMI1640; Lonza, Belgium) supplemented with 10% FBS (Thermo Fisher Scientific, Belgium) and 1% antibiotic‐antimycotic 100 × (Thermo Fisher Scientific), incubated for 20 min at 37°C, shaken vigorously for 15 s, and filtered through a 70 µm cell strainer (Greiner Bio‐One, Belgium). Repeat the procedure above, combining the two filtrates collected. The leftover tissue was digested in RPMI‐1640 with 1 mg/mL of collagenase type I (Sigma, USA), 60 U/mL DNase I (Invitrogen), and 10% FCS at 5.4g for 60 min at 37°C, and then filtered through a 70 µm cell strainer. To collect the cell pellet, the filtrate was centrifuged at 251.6g for 5 min and then blocked in 5% BSA for 1 h before staining. Neutrophils (CD11b^+^ Ly6G^+^), CD8^+^ T cells (CD3^+^CD8^+^) were used to characterize the cell types. BD Pharmingen provided APC Hamster anti‐mouse CD3^+^ for FACS (Cat. No.561826/553066). Thermo Fisher provided PerCP‐conjugated anti‐CD8^+^ (Product # MCD0831), anti‐mouse CD11b^+^ APC (Product # 17‐4031‐82), and FITC anti‐mouse Ly6G^+^ (Product # 11‐4031‐85) for FACS. A flow cytometer (FACS Calibur; BD Biosciences) with Cell Quest software was used to detect fluorescence (BD Biosciences, Canada).^36^

### 4.11 Mouse LPS ELISA

The kit is a double antibody, one‐step sandwich ELISA. To the coated wells precoated withLipopolysaccharides (LPS) antibody, the specimen, standard, and HRP‐labeled detection antibody are sequentially added, incubated, and washed thoroughly. The color is developed with the substrate TMB, which is converted to blue by the catalysis of peroxidase and to final yellow by acid. The shade of color is positively correlated with the LPS in the sample. The absorbance (OD) was measured at 450 nm using an enzyme meter, and the concentration of the sample was calculated.

### 4.12 Statistical analyses

Data were analyzed using GraphPad Prism 9.0 and SPSS statistical software (version 21.0). A one‐way analysis of variance was used to analyze the differences between groups, and a two‐tailed Student's *t*‐test was used to analyze the significance of the two groups.^37^ All data were presented as mean ± SEM. Statistical significance was determined at *p* < 0.05.

## AUTHOR CONTRIBUTIONS

Z.‐C. H. and H. Z. designed the outline of the paper. H. Z. revised this manuscript. J. X. and H. X. contributed equally to this work. J. X. and H. X. performed most of the experiments in this study. J. X. wrote the manuscript and prepared the figures, and H. X. helped her perfect the process. F. Y., Y. X., M. S., and F. C. helped with the molecular biology‐related experiments. H. X., F. C., and M. L. helped with the experiments using animals. All authors have read and approved the final version of this manuscript.

## CONFLICT OF INTEREST STATEMENT

The authors declare no conflict of financial interest.

## ETHICS STATEMENT

Animal welfare and experimental procedures were performed in strict accordance with high standard animal welfare and other related ethical regulations approved by the Nanjing University Animal Care and Use Committee. APPROVAL NUMBER for animal: IACUC‐2102008.

## Supporting information

Supporting Information

## Data Availability

The corresponding author will provide the datasets used and analyzed during the current work upon reasonable request. The raw sequence data reported in this paper have been deposited in the Genome Sequence Archive (Genomics, Proteomics & Bioinformatics 2021) in National Genomics Data Center (Nucleic Acids Res 2024), China National Center for Bioinformation/Beijing Institute of Genomics, Chinese Academy of Sciences (GSA: PRJCA026691) that are publicly accessible at https://ngdc.cncb.ac.cn/gsa.^38,39^
