# Supplementary material for: Different depths of food restriction and high‐fat diet refeeding in mice impact host obesity and metabolic phenotypes with correlative changes in the gut microbiota
Source: MedComm (2020). 2024 Jul 17;5(8):e641. doi: 10.1002/mco2.641 (PMC11253304; doi:10.1002/mco2.641)
Supplement: Supplementary file 1 — Supporting Information [file MCO2-5-e641-s001.docx]

# Different depths of food restriction and high-fat diet refeeding in mice impact host obesity and metabolic phenotypes with correlative changes in the gut microbiota

Jiaqi Xu^1,#^, Huangru Xu^1,#^, Feiyan Yang^1^, Yawen Xie^1^, Fangfang Cai^1^, Siyu Mao^1^, Min Lu^1^, Hongqin Zhuang^1,*^, Zi-Chun Hua^1,2,3,*^

*^1^The State Key Laboratory of Pharmaceutical Biotechnology and Department of Neurology of Nanjing Drum Tower Hospital, School of Life Sciences and The Affiliated Hospital of Nanjing University Medical School, Nanjing University, Nanjing 210023, China*

*^2^Faculty of Pharmaceutical Sciences, Xinxiang Medical University, Xinxiang 453002, China*

*^3^Changzhou High-Tech Research Institute of Nanjing University and Jiangsu TargetPharma Laboratories Inc., Changzhou 213164, P. R. China*

*^#^These authors contributed equally to this work.*

***Corresponding authors:**

Hongqin Zhuang, School of Life Sciences, Nanjing University, 163 Xianlin Blvd., Nanjing 210023, China. E-mail: [hqzhuang@nju.edu.cn](mailto:hqzhuang@nju.edu.cn)

Zi-Chun Hua, School of Life Sciences, Nanjing University, 163 Xianlin Blvd., Nanjing 210023, China. E-mail: [hzc1117@nju.edu.cn](mailto:hzc1117@nju.edu.cn)

**Supplementary materials**

**Table S1 Page3**

**Figure S1 Page4**

**Figure S2 Page6**

**Figure S3 Page7**

**Figure S4 Page8**

**Figure S5 Page9**

**Figure S6 Page10**

**Figure S7 Page11**

**Figure S8 Page13**

**Figure S9 Page14**

**Figure S10 Page16**

**Figure S11 Page18**

**Figure S12 Page19**

**Figure S13 Page21**

**Table S1.** **The primers of selected genes for real time PCR.**

| **Gene** | **Forward primer (5’-3’)** | **Reverse primer (5’-3’)** |
| --- | --- | --- |
| **Tnf-α** | CGTCAGCCGATTTGCTATCT | CGGACTCCGCAAAGTCTAAG |
| **Tgf-b** | GGAGGTACCGCCCGGCCCGC | GACAGCAATGGGGGTTCGGG |
| **Il-1b** | GAGAGCCGGGTGACAGTATC | TGACAAACTTCTGCCTGACG |
| **Il-6** | AGTTGCCTTCTTGGGACTGA | CAGAATTGCCATTGCACAAC |
| **Il-10** | CCAAGCCTTATCGGAAATGA | TTTTCACAGGGGAGAAATCG |
| **Actin** | GAGACCTTCAACACCCCAGC | ATGTCACGCACGATTTCCC |

**Figure S1. Body mass of the food-restricted young mice (6-week-old) refed with high-fat diet**


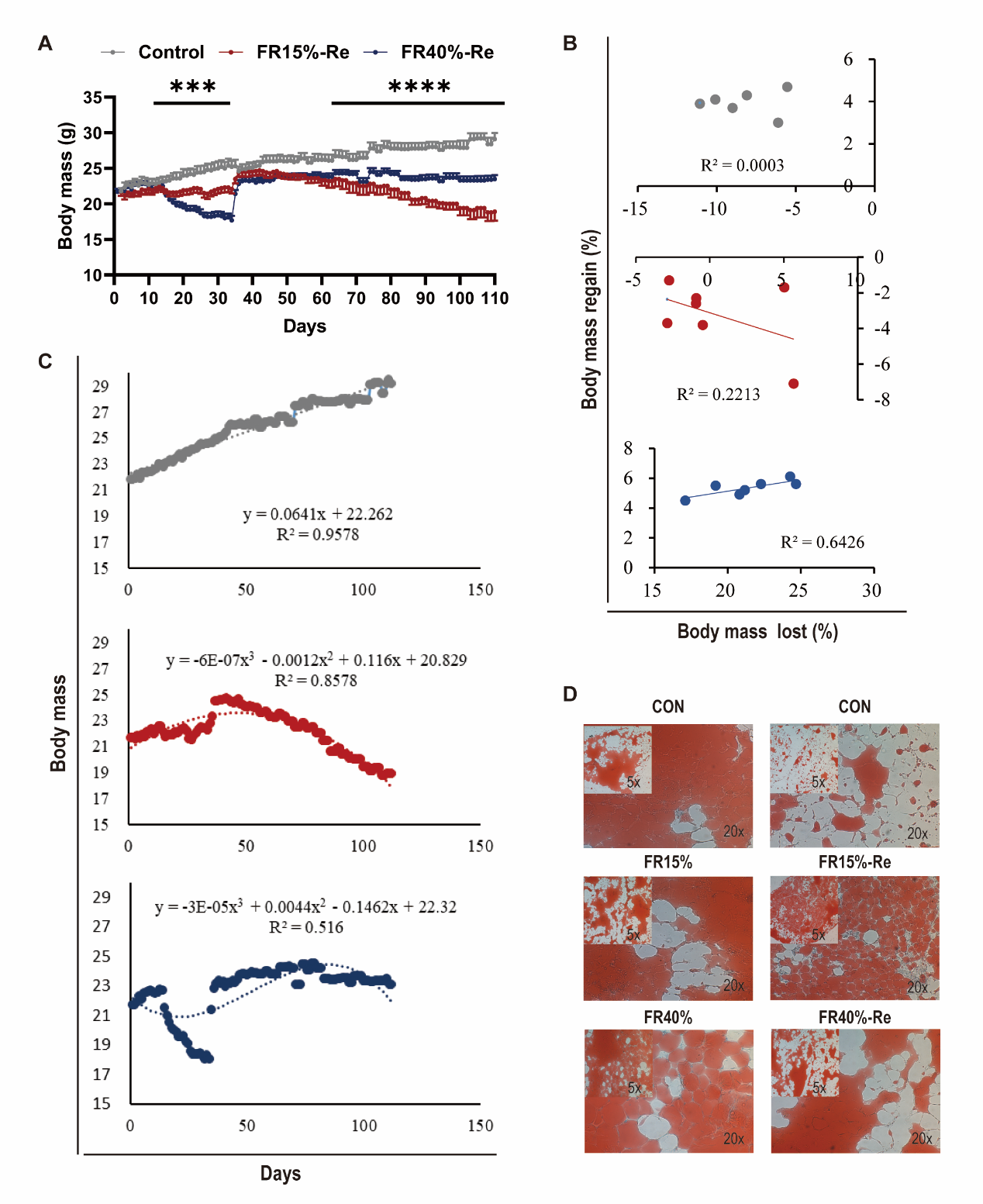


**Figure S1. Body mass of the food-restricted young mice (6-week-old) refed with high-fat diet.** **(A)** Body mass of the food-restricted young mice refed with high-fat diet. **(B)** Correlations between body mass lost during 2 weeks of food restriction and body mass regain of young mice refed with high-fat diet for 10 weeks. **(C)** The functional relationship and fitting curve for the three groups’ changes in body mass. **(D)** White adipose tissues were stained with Oil red O. CON, the young mice were fed ad libitum throughout the experiment; FR15%-Re, FR40%-Re, the young mice were restricted by 15% and 40% of ad libitum food intake, respectively, for 2 weeks and followed by high-fat diet refeeding for 10 weeks. Data were represented as mean ± SEM. n = 6 mice in each group. **P < 0.05, **P < 0.01, ***P < 0.001, ****P < 0.0001.*


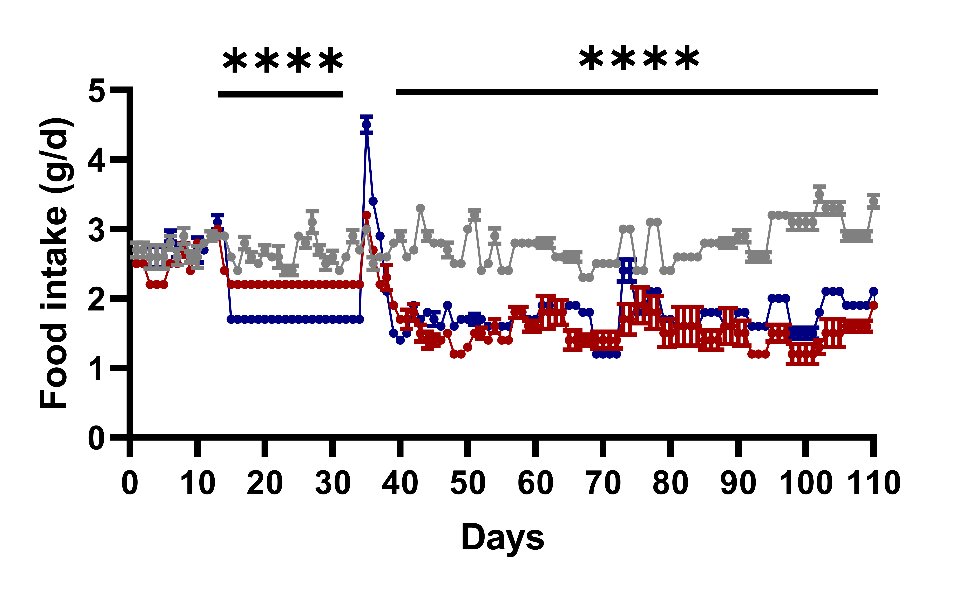
**Figure S2.** **Food intake of the food-restricted young mice (6-week-old) refed with high-fat diet**

**Figure S2.** **Food intake of the food-restricted young mice (6-week-old) refed with high-fat diet.** CON, the young mice were fed ad libitum throughout the experiment; FR15%-Re, FR40%-Re, the young mice were restricted by 15% and 40% of ad libitum food intake, respectively, for 2 weeks and followed by high-fat diet refeeding for 10 weeks. Data were represented as mean ± SEM. n = 6 mice in each group. **P < 0.05, **P < 0.01, ***P < 0.001, ****P < 0.0001.*

**Figure S3.** **Body mass change, body fat mass and body fat content detections of the food-restricted young mice refed with high-fat diet**

**
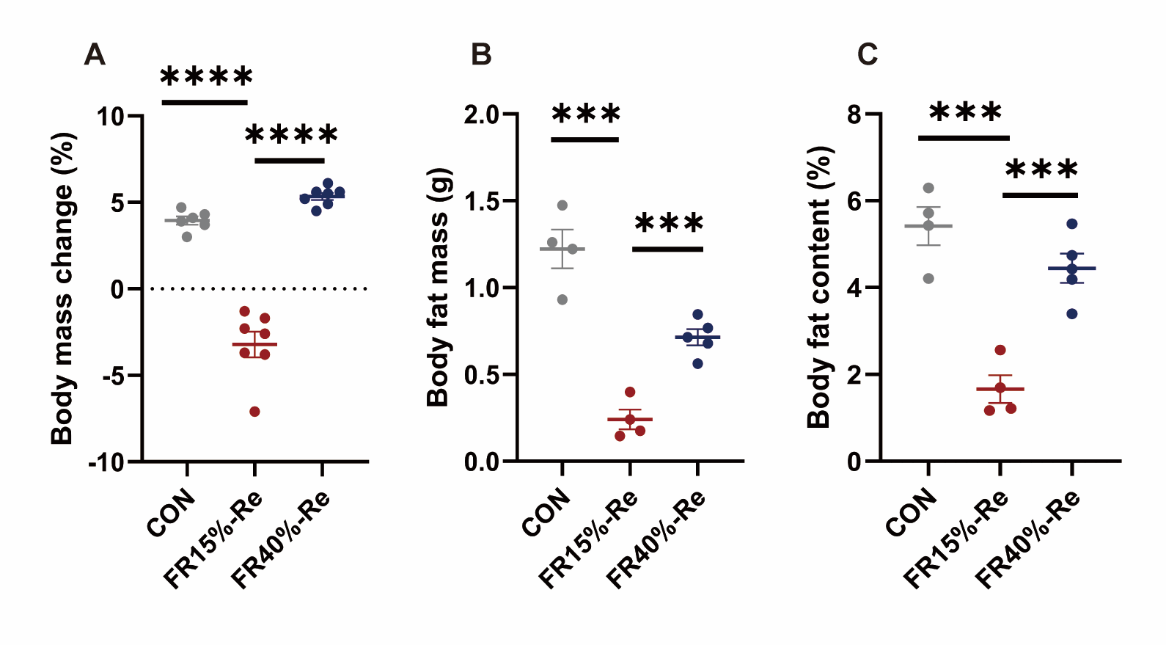
**

**Figure S3.** **Body mass change, body fat mass and body fat content detections of the food-restricted young mice refed with high-fat diet. (A)** Body mass change. **(B)** Body fat mass. **(C)** Body fat content. CON, the young mice were fed ad libitum throughout the experiment; FR15%-Re, FR40%-Re, the young mice were restricted by 15% and 40% of ad libitum food intake, respectively, for 2 weeks and followed by high-fat diet refeeding for 10 weeks. Data were represented as mean ± SEM. n = 6 mice in each group. **P < 0.05, **P < 0.01, ***P < 0.001, ****P < 0.0001.*

**
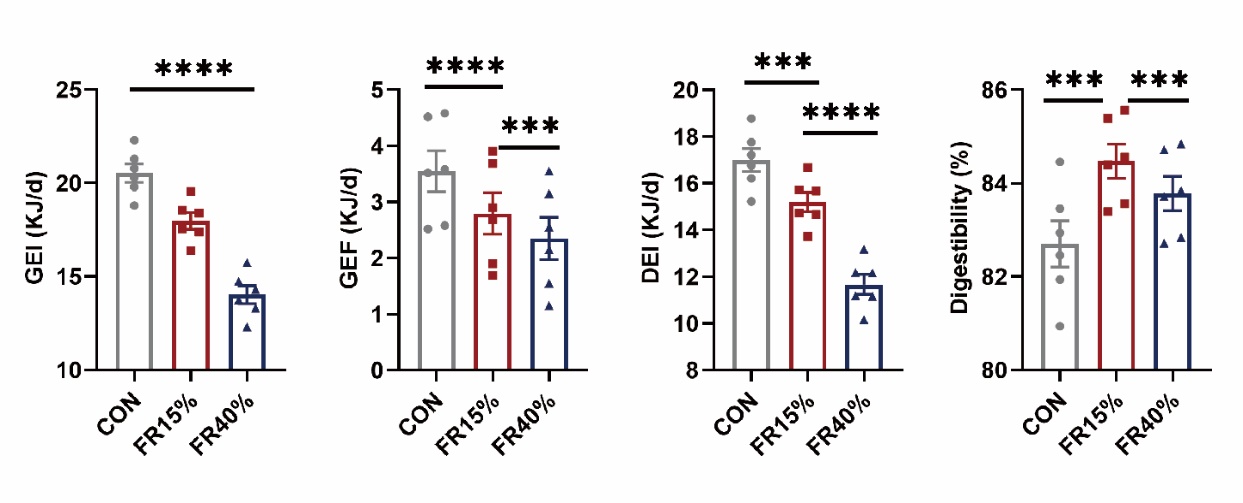
Figure S4.** **Gross energy intake (GEI), gross energy of feces (GEF), digestive energy intake (DEI) and digestibility detections of the food-restricted young mice7**

**Figure S4.** **Gross energy intake (GEI), gross energy of feces (GEF), digestive energy intake (DEI) and digestibility** **detections of the food-restricted young mice.** Gross energy intake. Gross energy of feces. Digestive energy intake. Digestibility. CON, the young mice were fed ad libitum throughout the experiment; FR15%-Re, FR40%-Re, the young mice were restricted by 15% and 40% of ad libitum food intake, respectively, for 2 weeks and followed by high-fat diet refeeding for 10 weeks. Data were represented as mean ± SEM. n = 6 mice in each group. **P < 0.05, **P < 0.01, ***P < 0.001, ****P < 0.0001.*

**
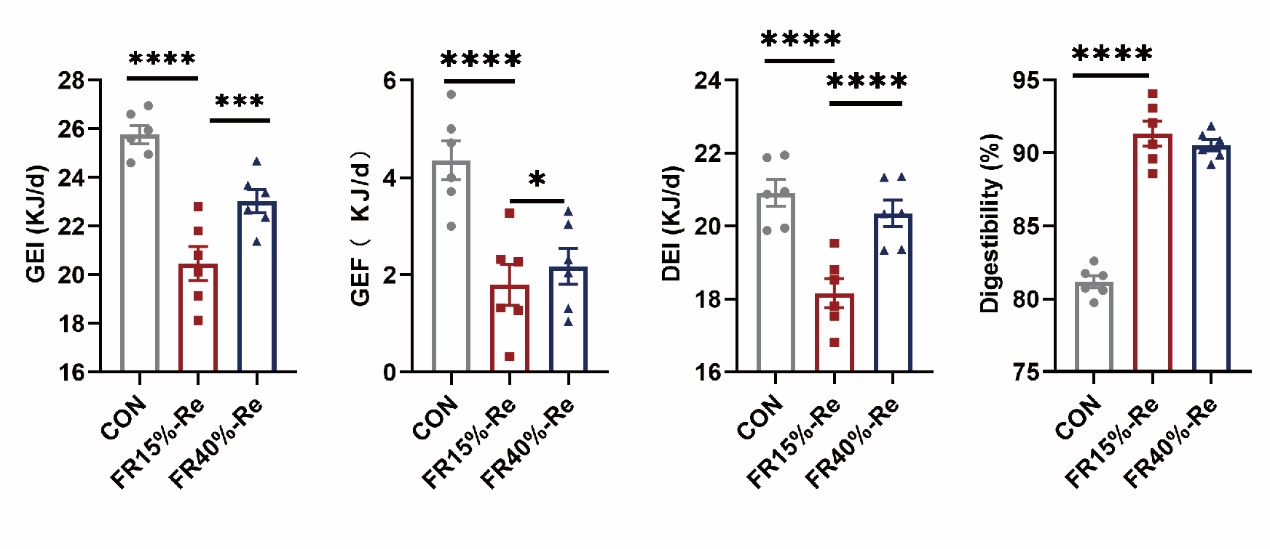
Figure S5.** **GEI, GEF, DEI and digestibility detections of the food-restricted young mice refed with high-fat diet**

**Figure S5. GEI, GEF, DEI and digestibility** detections **of the food-restricted young mice refed with high-fat diet.** Gross energy intake. Gross energy of feces. Digestive energy intake. Digestibility. CON, the young mice were fed ad libitum throughout the experiment; FR15%-Re, FR40%-Re, the young mice were restricted by 15% and 40% of ad libitum food intake, respectively, for 2 weeks and followed by high-fat diet refeeding for 10 weeks. Data were represented as mean ± SEM. n = 6 mice in each group. **P < 0.05, **P < 0.01, ***P < 0.001, ****P < 0.0001.*

**
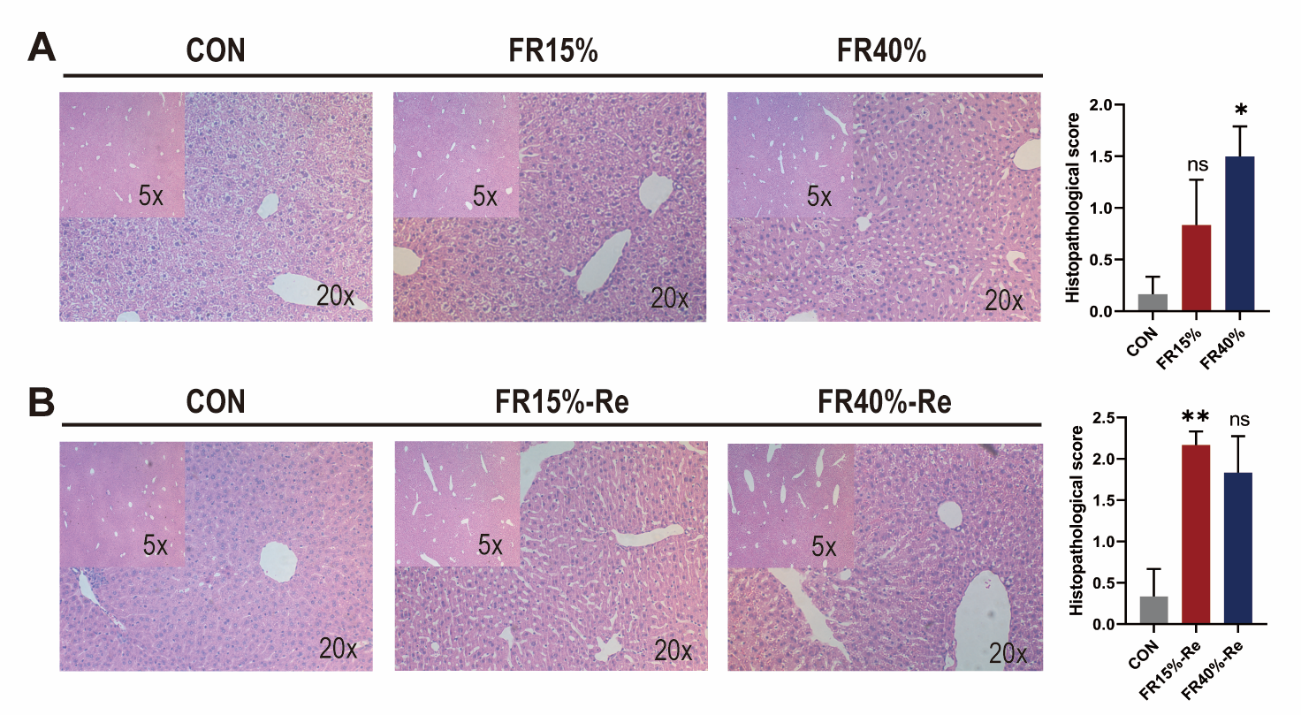
** **Figure S6.** **Observations on the change of liver tissues morphology under food restriction and refeeding**

**Figure S6. Observations on the change of liver tissues morphology under food restriction and refeeding. (A)** H&E-stained paraffin slices of liver tissues from three FR groups. Bars indicate 100 μm. Histopathological scores of liver tissues sections (histological score = damage × width of lesions). **(B)** H&E-stained paraffin slices of liver tissues from three FR-Re groups. Bars indicate 100 μm. Histopathological scores of liver tissues sections (histological score = damage × width of lesions). CON, the adult mice were fed ad libitum throughout the experiment; FR15%-Re, FR40%-Re, the adult mice were restricted by 15% and 40% of ad libitum food intake, respectively, for 2 weeks and followed by high-fat diet refeeding for 6 weeks. Data were represented as mean ± SEM. n = 6 mice in each group. **P < 0.05, **P < 0.01, ***P < 0.001, ****P < 0.0001.*

**Figure S7. Food restriction and refeeding alter the fecal microbiota of young mice**

**
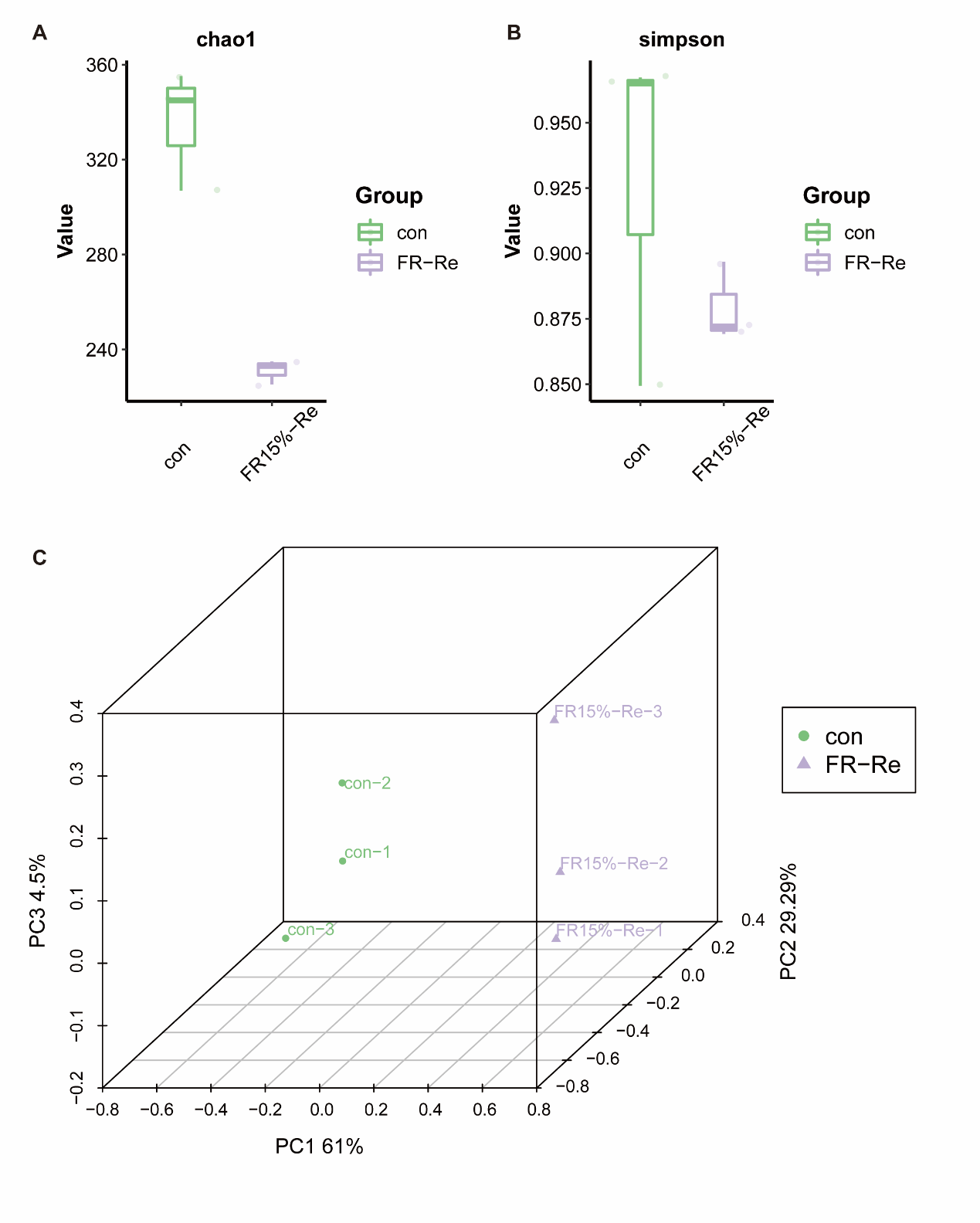
**

**Figure S7. Food restriction and refeeding alter the fecal microbiota of young mice. (A)** Alpha diversity changed during the refeeding period. The Chao1 index levels decreased during the refeeding period. **(B)** The Simpson index levels decreased during the food-restriction period. **(C)** Beta diversity changed during the refeeding period. CON, the young mice were fed ad libitum throughout the experiment; FR15%-Re, the young mice were restricted by 15% of ad libitum food intake, respectively, for 2 weeks and followed by high-fat diet refeeding for 10 weeks. Data were represented as mean ± SEM. n = 6 mice in each group. **P < 0.05, **P < 0.01, ***P < 0.001, ****P < 0.0001.*

**Figure S8. Food restriction and refeeding impact fecal microbiota composition**

**
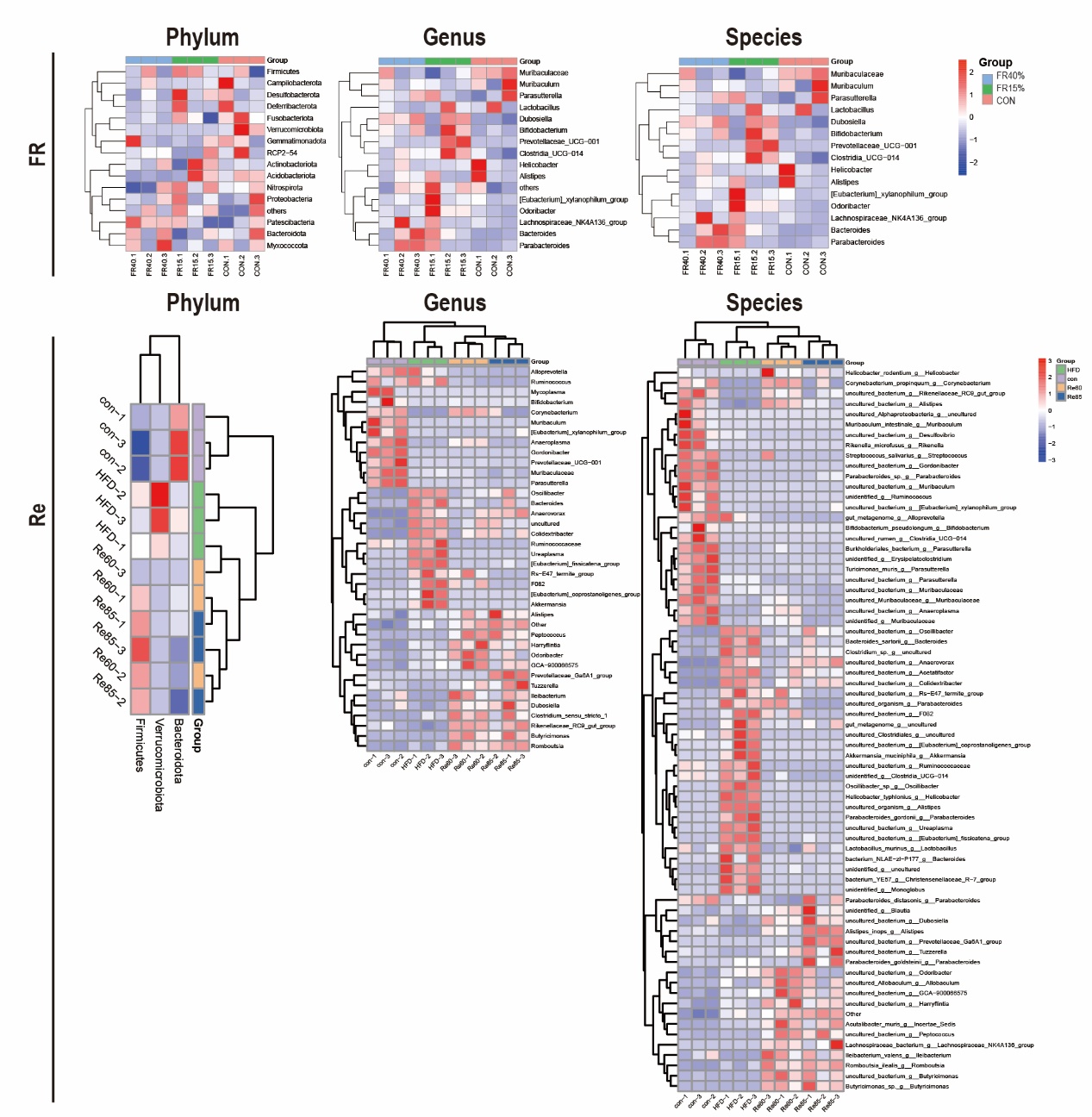
**

**Figure S8. Food restriction and refeeding impact fecal microbiota composition.**

Community feature cluster maps for the two FR groups and FR-Re groups. CON, the animals were fed ad libitum throughout the experiment; HFD, the animals were fed 60% high-fat chow throughout the experiment; FR15%-Re, FR40%-Re, the animals were restricted by 15% and 40% of ad libitum food intake, respectively, for 2 weeks and followed by high-fat diet refeeding for 6 weeks. Data were represented as mean ± SEM. n = 6 mice in each group. *P < 0.05, **P < 0.01, ***P < 0.001, ****P < 0.0001.

**Figure S9. Food restriction and refeeding impact fecal microbiota composition of young mice**

**
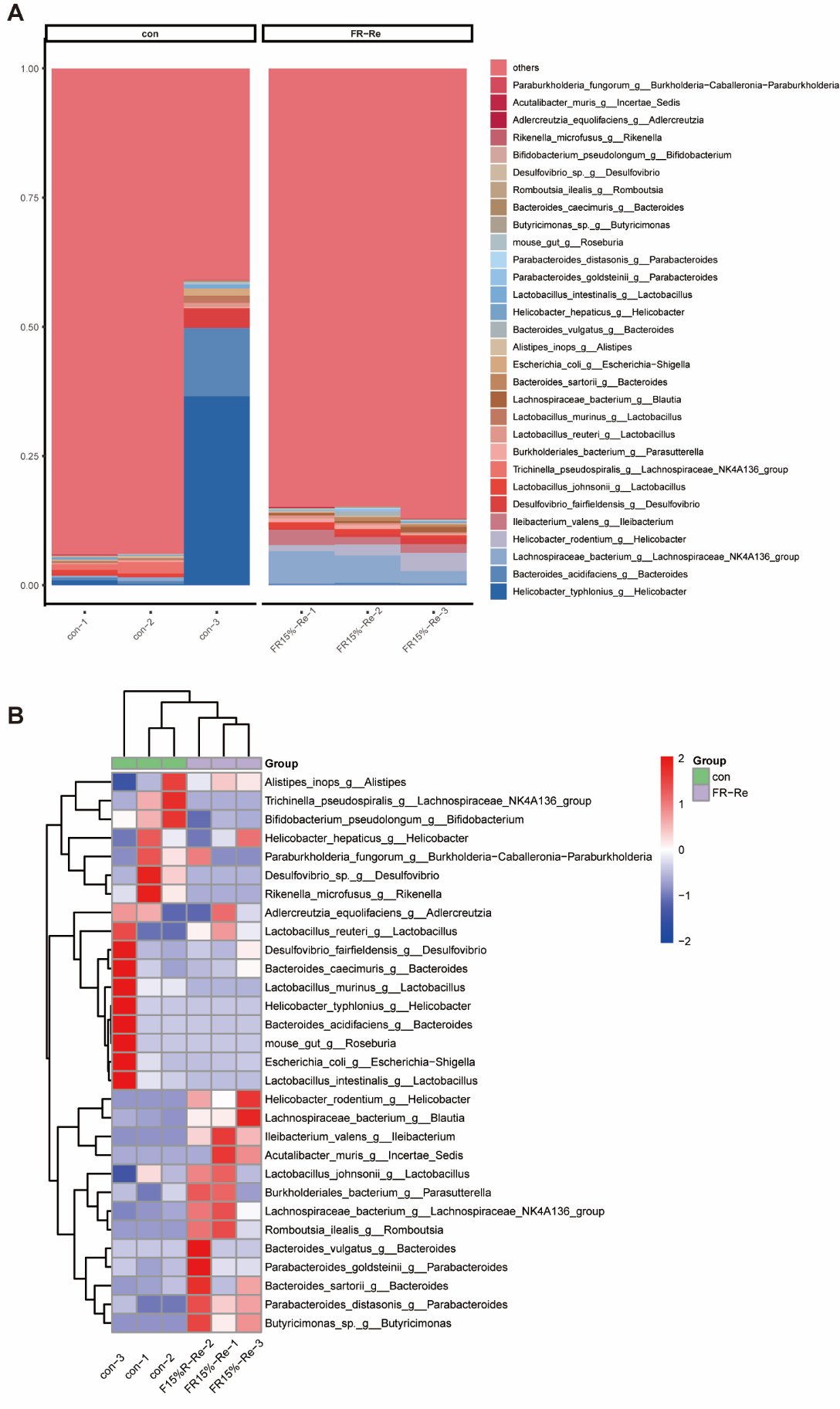
**

**Figure S9. Food restriction and refeeding impact fecal microbiota composition of young mice. (A)** Microbiota species composition in the control and FR15%-Re groups. **(B)** Community feature cluster maps for the two groups. CON, the young mice were fed ad libitum throughout the experiment; FR15%-Re, the young mice were restricted by 15% of ad libitum food intake, respectively, for 2 weeks and followed by high-fat diet refeeding for 10 weeks. Data were represented as mean ± SEM. n = 6 mice in each group. Positive correlations are in red and negative correlations are in green.

**Figure S10.** **Differences in species between the control and the FR15%-Re adult mice**

**
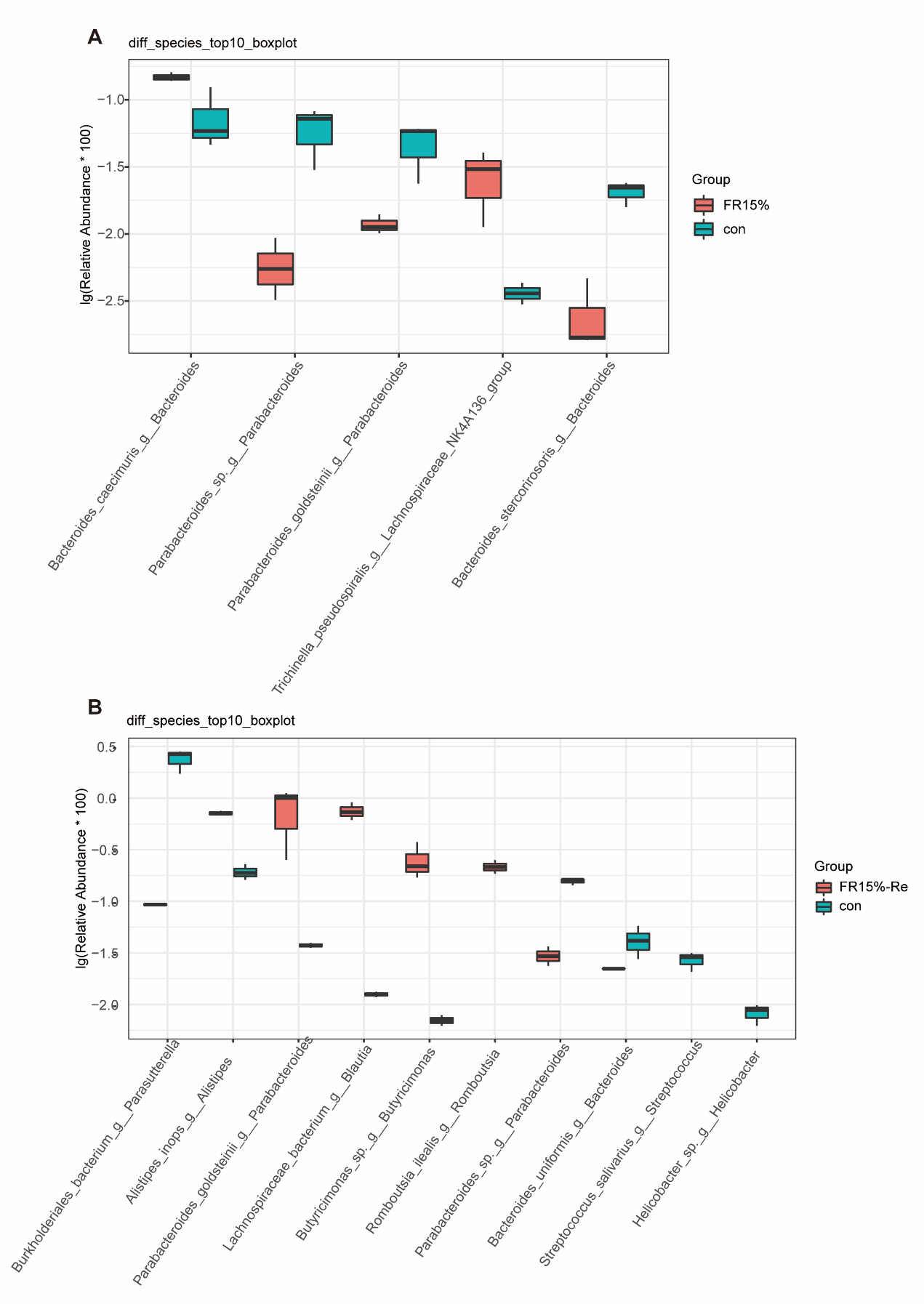
**

**Figure S10. Differences in species between control and FR15%-Re adult mice. (A)** Top ten species level microbiota differences between the control and FR15% adult mice. **(B)** Top ten species level microbiota differences between the control and FR15%-Re adult mice. CON, the adult mice were fed ad libitum throughout the experiment; FR15%-Re, the adult mice were restricted by 15% of ad libitum food intake, respectively, for 2 weeks and followed by high-fat diet refeeding for 6 weeks. Data were represented as mean ± SEM. n = 6 mice in each group.

**Figure S11. Differences in species between control and FR15%-Re young mice**

**
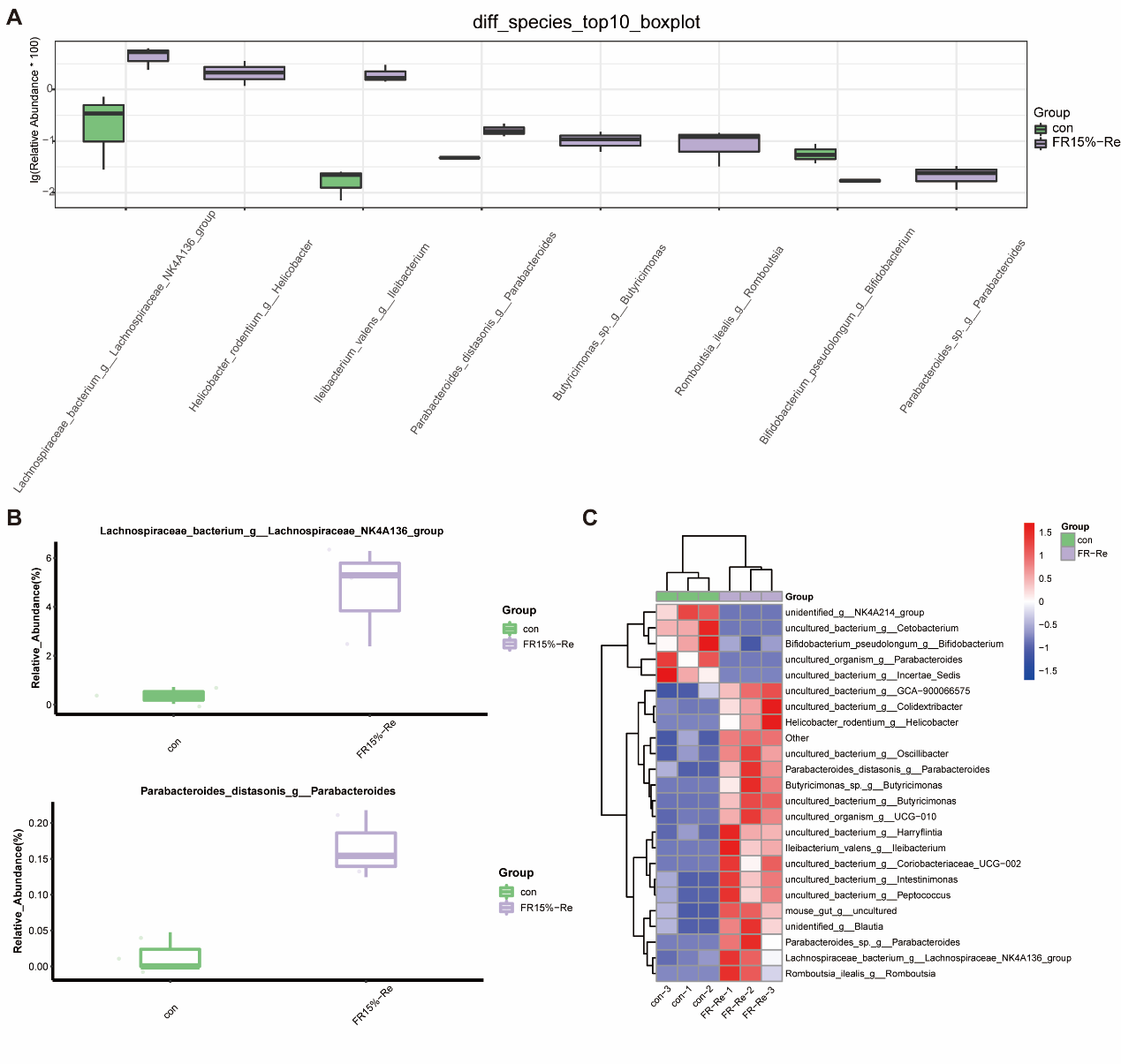
 Figure S11. Differences in species between control and FR15%-Re young mice. (A)** Top ten species level microbiota differences between the two groups. **(B)** Two significant species level microbiota differences between the two groups. **(C)** The microbiota is ranked by species-level differences in association between the two groups. CON, the young mice were fed ad libitum throughout the experiment; FR15%-Re, the young mice were restricted by 15% of ad libitum food intake, respectively, for 2 weeks and followed by high-fat diet refeeding for 10 weeks. Data were represented as mean ± SEM. n = 6 mice in each group. Positive correlations are in red and negative correlations are in green.

**Figure S12.** **Compared with species differences between food restriction and refeeding mice**

**
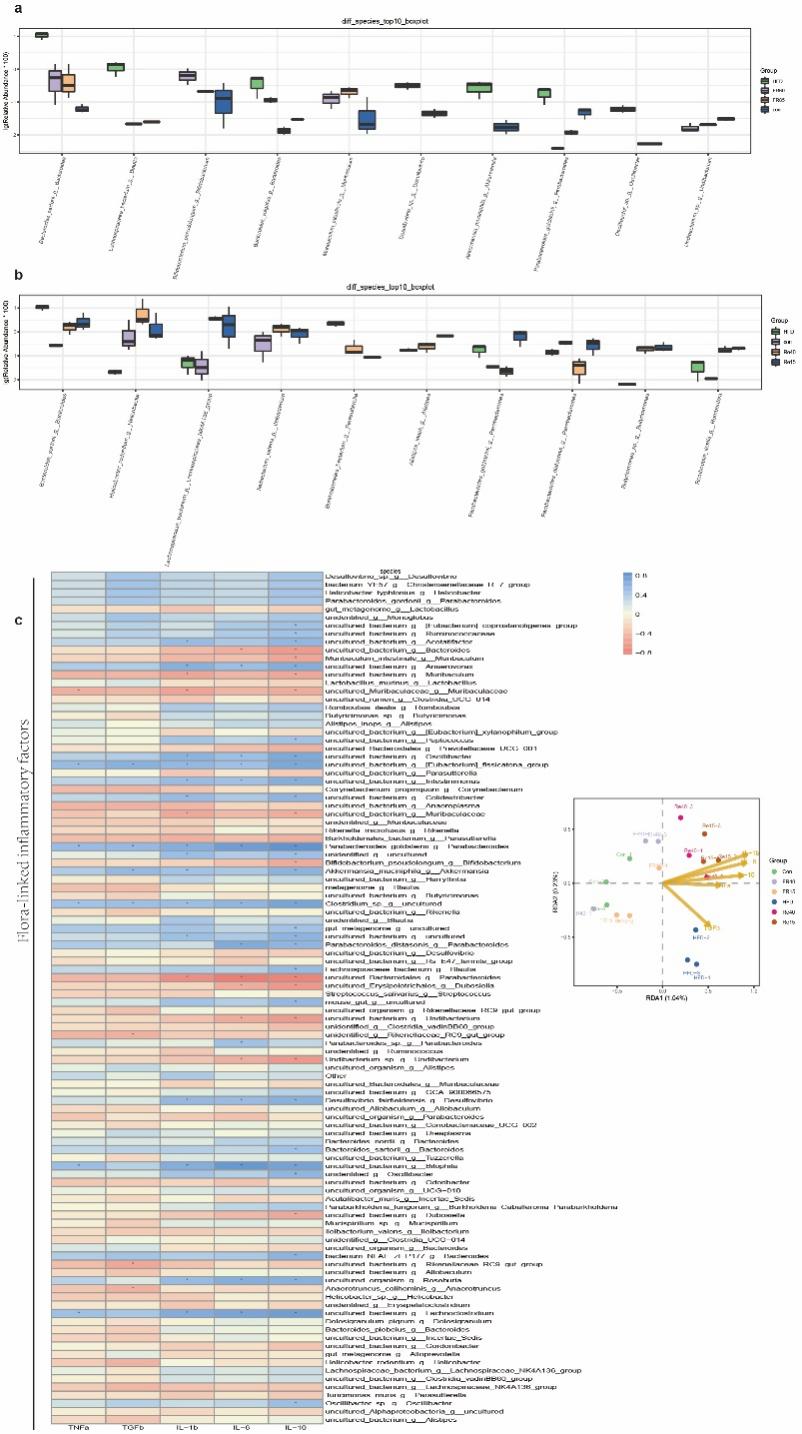
**

**Figure S12.** **Compared with species differences between food restriction and refeeding mice**

**(A) Top ten species-level microbiota differences between the two FR groups. (B) Top ten species level microbiota differences between the two FR-Re groups. (C) Association of microbiota with inflammatory factors between control group, high-fat group, 2 food-restricted groups and 2 heavy-feeding groups CON, the animals were fed ad libitum throughout the experiment; HFD, the animals were fed 60% high-fat chow throughout the experiment; FR15%-Re, FR40%-Re, the animals were restricted by 15% and 40% of ad libitum food intake, respectively, for 2 weeks and followed by high-fat diet refeeding for 6 weeks. Data were represented as mean ± SEM. n = 6 mice in each group. Positive correlations are in red and negative correlations are in green.**

**Figure S13. The microbiota correlation analysis**

**
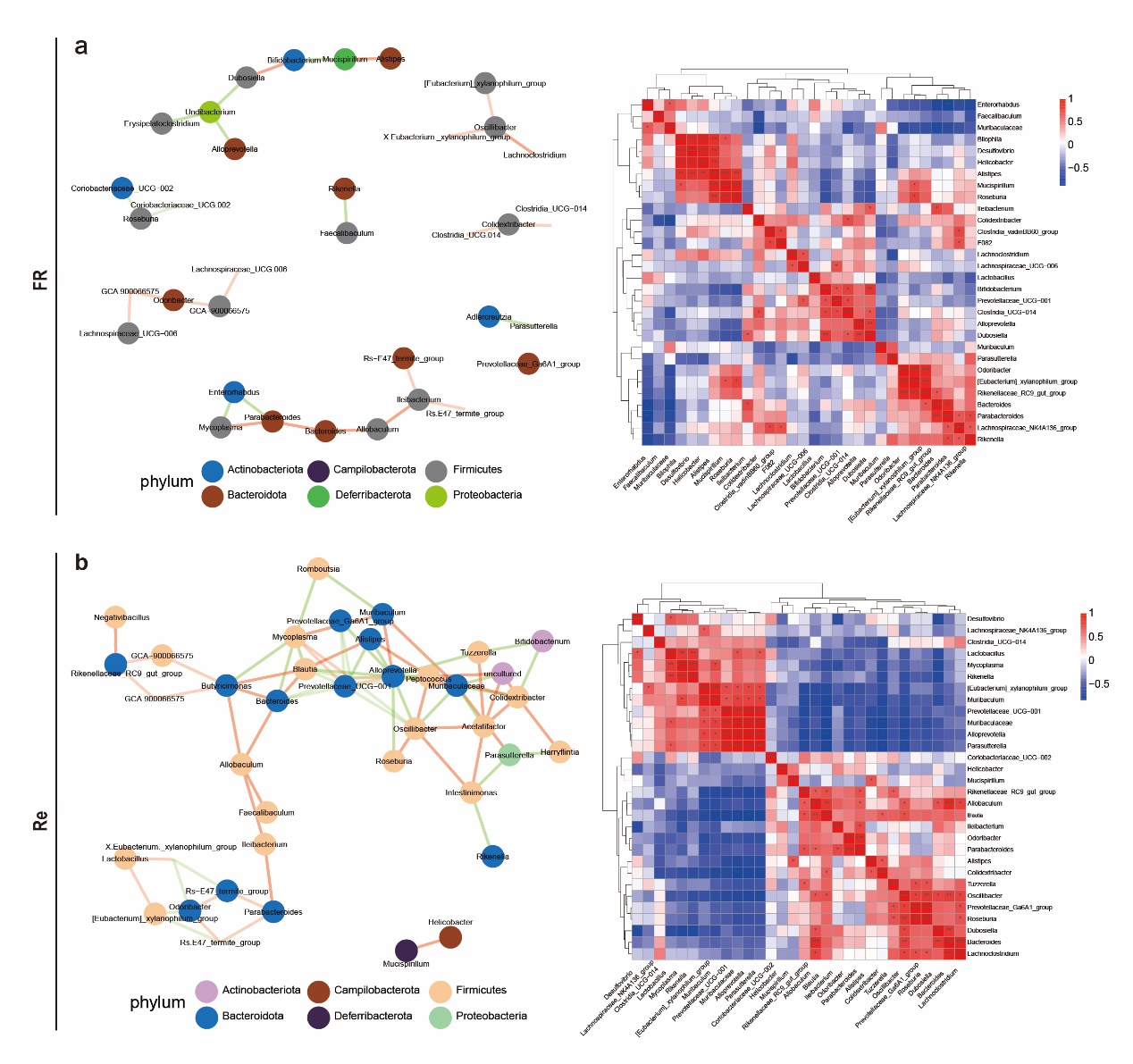
**

**Figure S13. The microbiota correlation analysis**

**(A)** The two FR groups' microbiota correlation analysis. **(B)** The two FR-Re groups' microbiota were analyzed for correlation. CON, the animals were fed ad libitum throughout the experiment; HFD, the animals were fed 60% igh-fat chow throughout the experiment; FR15%-Re, FR40%-Re, the animals were restricted by 15% and 40% of ad libitum food intake, respectively, for 2 weeks and followed by high-fat diet refeeding for 6 weeks. Data were represented as mean ± SEM. n = 6 mice in each group. Positive correlations are in red and negative correlations are in green.
